# HOXA9 orchestrates EMT and metastasis in oral cancer via transcriptional activation of vimentin and β-catenin signaling

**DOI:** 10.1038/s41419-026-08664-7

**Published:** 2026-03-28

**Authors:** U. Sangeetha Shenoy, Divya Adiga, Dhanraj Salur Basavarajappa, Naveena A. N. Kumar, Adarsh Kudva, Ajaikumar B. Kunnumakkara, Keith D. Hunter, Shama Prasada Kabekkodu, Raghu Radhakrishnan

**Affiliations:** 1 Department of Cell and Molecular Biology, Manipal School of Life Sciences, Manipal Academy of Higher Education, Manipal, Karnataka India; 2 Department of Surgical Oncology, Kasturba Medical College and Hospital, Manipal Academy of Higher Education, Manipal, Karnataka India; 3 Department of Oral and Maxillofacial Surgery, Kasturba Medical College and Hospital, Manipal Academy of Higher Education, Manipal, Karnataka India; 4 Cancer Biology Laboratory, Department of Biosciences and Bioengineering, Indian Institute of Technology, Guwahati, Assam India; 5 Liverpool Head and Neck Centre, Molecular and Clinical Cancer Medicine, University of Liverpool, Liverpool, UK; 6 Department of Oral and Maxillofacial Pathology and Oral Microbiology, Manipal College of Dental Sciences, Manipal, Manipal Academy of Higher Education, Manipal, Karnataka India; 7 Academic Unit of Oral and Maxillofacial Medicine and Pathology, School of Clinical Dentistry, University of Sheffield, Sheffield, UK; 8 Oral Biology and Pathology, Oman Dental College, Muscat, Oman

**Keywords:** Oral cancer, Oncogenesis, Cell signalling

## Abstract

Identifying biomarkers of epithelial‒mesenchymal transition (EMT) and metastasis play a decisive role in the prognosis and clinical management of Oral cancer (OC). Homeobox A9 (HOXA9), an imperative regulator of embryogenesis and endothelial cell proliferation, is aberrantly expressed in several malignancies, including OC. To date, HOXA9-mediated molecular mechanisms and their functional roles in EMT and OC metastasis remain poorly understood. In this study, we observed significant upregulation of HOXA9 in OC samples with lymph node-positive stage and higher histological grade. Notably, we demonstrated that retroviral-mediated knockdown of HOXA9 impairs proliferation, migration, and invasion while promoting apoptosis and cell cycle arrest. A substantial reduction in tumor volume and diminished lung metastasis was observed in nude mice receiving HOXA9-knockdown cells. Transcriptomic analysis of HOXA9-depleted cells revealed the downregulation of multiple pathways, with the most significant being “pathways in cancer”. We further demonstrated that HOXA9 transcriptionally activates *VIM* to promote EMT and it also facilitates β-catenin nuclear translocation, which subsequently activates downstream target genes driving Wnt/β-catenin cascade. Our study also unveils HOXA9-driven molecular interplay in which HOXA9-dependent transcriptional activation of *VIM* triggers Wnt/β-catenin cascade in OC. Furthermore, we demonstrated that hypomethylated CpGs at *HOXA9* promoter (spanning −4201bp to −3574bp upstream of TSS) in stage III/IV tumors showed a significant inverse correlation between hypomethylation and gene upregulation. Consistently, analysis of the same distal promoter region revealed the enrichment of activating histone modifications, further supporting its transcriptional permissive state. Collectively, our study uncovers a novel mechanism by which epigenetically altered *HOXA9* serves as a potential inducer of EMT through regulation of VIM/Wnt-β-catenin/EMT signaling axis. These findings signify HOXA9 as a promising biomarker in OC and targeting HOXA9 could be an effective strategy to improve clinical outcome of patients diagnosed with advanced stages.

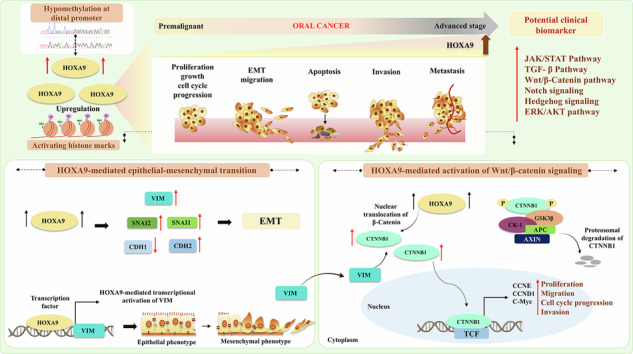

## Introduction

Oral cancer (OC) is a major global clinical burden, causing significant morbidity and mortality and ranking 16th among all cancers in terms of incidence rate [1]. According to GLOBOCAN 2022, the worldwide incidence of cancers of the lip and oral cavity was reported to be 389,846 newly reported cases and 188,438 deaths [1]. The 5-year prevalence rate for OC is 62.4% in Asian countries [1, 2]. This is due to delays in clinical diagnosis and the absence of reliable biomarkers. Recent reports have suggested that homeobox (HOX) cluster genes could serve as novel biomarkers for detecting OC progression [3–6]. These genes were initially discovered while studying mutations in the fruit fly *Drosophila melanogaster* and are conserved across species owing to evolutionary processes. These genes emerged from an ancestral HOX gene cluster via a series of duplications and subsequent divergence [7, 8]. There are 39 HOX genes in mammals that are arranged into 4 distinct HOX clusters occupying unique chromosomal positions [9, 10]. Upon translation, they serve as transcription factors to specify the body axis, skeletal shape, and organ growth during embryogenesis in a tissue-specific pattern. The “homeodomain” region allows them to specifically bind and regulate the target genes involved in crucial signaling pathways.

Among 39 HOX genes, the *HOXA9* gene has been widely explored, particularly in hematological and solid cancers [11, 12]. Under typical physiological circumstances, HOXA9 is recognized as a vital controller of morphogenesis [13, 14] and embryo implantation [15]. and also functions as a master regulator of hematopoiesis [16]. and endothelial cell regulation [17]. This is attributed to the HOXA9-driven transcriptional activation of target genes, which are highly enriched in these biological processes. Consequently, studies have demonstrated that aberrant expression of *HOXA9* disrupts the overall transcriptional landscape and associated biological events, causing developmental abnormalities in multiple diseases, including cancer [14, 18–20]. *HOXA9* dysregulation has been implicated in the cancer-related biological features, including epithelial‒mesenchymal transition (EMT) and metastasis in multiple malignancies [21–23]. Since EMT is a multidimensional phenomenon, regulated by crucial signaling pathways, and serves as a fundamental driver to metastasis, recurrence, and drug resistance, it has drawn considerable interest. Previous studies have demonstrated the potential role of HOXA9 as a transcriptional activator of key signaling molecules involved in EMT [23–27]. While HOXA9 known to function as a critical regulator of cellular proliferation and differentiation at normal physiological conditions, its involvement in EMT and OC metastasis has not yet been systematically investigated. Due to the complexity of *HOXA9* regulation, its interaction with other transcription factors and potential crosstalk with signaling molecules in cancer, defining its precise role presents significant challenges for researchers. Hence, in vitro studies involving cell lines and clinical samples and in vivo studies using animal mouse models have enabled researchers to study EMT and metastasis in different cancer types, including OC [21, 28, 29].

Aberrant expression of *HOXA9* is linked to epigenetic alterations at *HOXA9* locus across cancers [21, 30–36]. Uchida et al. have reported that *HOXA9* promoter hypermethylation is associated with increased metastasis risk [37]. and its detection in the salivary rinses of OC patients serves as a promising non-invasive biomarker in OC [38]. However, its association with *HOXA9* upregulation in OC remains unexplored. Prior studies show that hypomethylated promoter enriched with H3K27Ac and H3K4Me3 activates oncogenes in cancer [39, 40] and *HOXA9* upregulation in prostate cancer (PCa) is driven by H3K4Me3 deposition [41]. In this context, we identified functional promoter and analyzed OC samples to evaluate promoter DNA methylation status and associated histone modifications. Hence, our study aimed to unravel the clinical relevance of *HOXA9*, determine the regulation and biological implications of *HOXA9* dysregulation and investigate the HOXA9-mediated signaling pathways contributing to EMT and OC metastasis.

In the present study, we observed a significant upregulation of *HOXA9* in lymph-node positive-OC tissues, which may be attributed to hypomethylation of the distal promoter, enriched with activating histone marks. This is the first study to identify HOXA9 as an oncogenic driver and a molecular orchestrator of EMT in OC via regulation of VIM/Wnt-β-catenin/EMT signaling axis. Targeting HOXA9 could be an effective strategy to improve the clinical outcomes of patients diagnosed with advanced stages.

## Results

### HOXA9 is significantly upregulated in OC

**Fig. 1:**
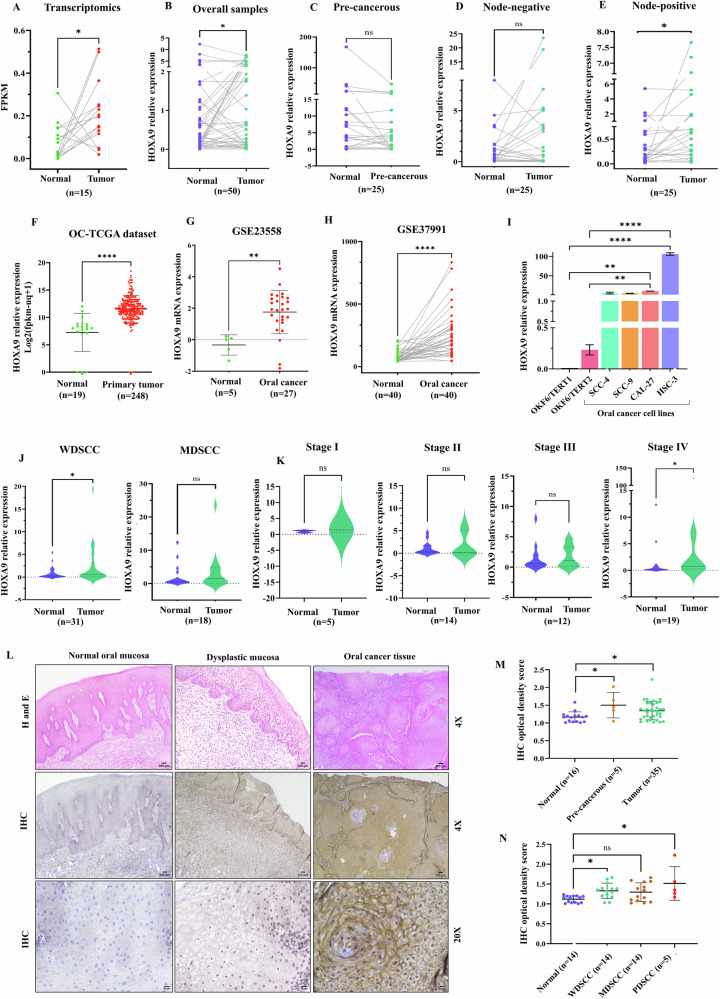
*HOXA9* is differentially expressed in OC. **A** Connecting dot plot representing the RNA sequencing data, showing *HOXA9* upregulation in OC (*n* = 15) patients, compared with matched normal. **B** qRT-PCR analysis validating the upregulation of *HOXA9* in OC samples (*n* = 50), compared with matched normal controls. There was no significant difference in the expression across **C** PC samples and **D** NN samples. **E** Connecting dot plot of qRT-PCR data revealed significant upregulation of *HOXA9* in the NP samples (*n* = 25). **F** Extensive analysis of OC-TCGA datasets revealed increased *HOXA9* mRNA levels in primary tumor samples (*n* = 248), compared to the solid tissue normal samples (*n* = 19). Analysis of the GEO dataset **G** GSE23558 has also shown *HOXA9* overexpression in OC samples (*n* = 27), compared to the samples collected from healthy individuals (*n* = 5). **H** GSE37991 also exhibited *HOXA9* overexpression in OC samples (*n* = 40), compared to matched normal samples. **I** *HOXA9* expression in OC cell lines, compared to immortalized normal oral keratinocytes. **J** Expression of *HOXA9* in WDSCC and MDSCC samples, showing significant upregulation of *HOXA9* in WDSCC samples. **K** Expression of *HOXA9* in different clinical stages (Stages I–IV). High expression of *HOXA9* was observed in stage IV cases of OC. **L** Representative H&E images of normal mucosa, dysplastic mucosa and OC tissues (upper panel). The lower panel shows the corresponding images of IHC staining with HOXA9 antibody. OC samples exhibited high positive staining in nucleus and cytoplasm, compared to normal and dysplastic oral tissues. **M** Quantitative analysis of IHC data from the clinical progression model of OC, confirming the highest optical density score in PC and OC samples. **N** Quantitative analysis of IHC data in different histological grades of OC. A higher IHC optical density score in WDSCC and PDSCC samples indicates the increased HOXA9 protein levels with progressive histological grades. The asterisk represents statistical significance (*P < 0.05).

Transcriptome data analysis revealed differential expression of *HOXA9* in tumor samples (*n* = 15; Fig. 1A). Validation with qRT‒PCR analysis showed significant upregulation of *HOXA9* mRNA in 28 out of 50 OC samples (Fig. 1B). Nevertheless, there was no notable difference in *HOXA9* expression in pre-cancerous (PC) (Fig. 1C) and node-negative (NN) samples (Fig. 1D). In contrast, *HOXA9* was markedly elevated (15 out of 25 samples), particularly in the advanced stage tumors with positive lymph node metastasis (NP-node-positive) suggesting its potential involvement in OC progression (Fig. 1E). These findings aligned with the OC-TCGA (Fig. 1F) and GEO datasets (Fig. 1G, H). Consistently, a similar trend was observed in OC cells in which *HOXA9* expression was higher than that in normal oral keratinocytes (Fig. 1I).

### Clinical significance of HOXA9 in OC

Differential *HOXA9* expression in relation to clinical features is summarized in Table 1. The expression of *HOXA9* was highly correlated with the site of the tumor. The analysis revealed that 57.7% of the buccal mucosal tissues and 61.53% of tongue tissues presented upregulation (FC ≥ 1). This indicates upregulation of *HOXA9* in OC, regardless of the tumor site within the oral cavity. Analysis of gene expression across different histological grades, revealed that *HOXA9* mRNA was highly upregulated in WDSCC (Fig. 1J), whereas no significant differential expression was observed in MDSCC. However, expression levels of *HOXA9* continue to increase in successive stages of OC, with the highest and most significant in stage IV samples (Fig. 1K). Notably, its marked upregulation in lymph node-positive tumors, across different histological grades, including WDSCC and stage IV cases suggests its potential role in tumor invasion, metastasis, and therapy resistance. Underscoring the clinical relevance of *HOXA9*, our observations could help the clinicians identify disease progression to an advanced stage.

**Table 1:** Differential expression of *HOXA9* in relation to clinicopathological features of the OC samples, analyzed via a qRT-PCR dataset.

	Oral cancer–Clinical samples *n* (%)		
	HOXA9 expression FC ≥ 1	HOXA9 expression FC < 1	*P*-value (between normal and tumor)
Risk factors			
With habits	20 (58.82)	14 (41.17)	0.1024
Without habits	9 (56.25)	7 (43.75)	0.2293
Tumor site			
Buccal mucosa	15 (57.7)	11 (42.3)	0.0755
Gingivo buccal sulcus	3 (75)	1 (25)	0.250
Tongue	8 (61.53)	5 (38.46)	0.677
Alveolus	2 (66.66)	1 (33.3)	0.750
Maxilla	-	-	-
Floor of mouth	-	-	-
Pre-malignant lesions	11 (44)	14 (56)	0.0501
Nodal status			
Node-negative	13 (52)	12 (48)	0.2872
Node-positive	15 (60)	10 (40)	0.0275
Pathological stages			
Stage I	4 (80)	1 (20)	0.1875
Stage II	8 (57.14)	6 (42.85)	0.5416
Stage III	5 (41.66)	7 (58.33)	0.7163
Stage IV	12 (63.15)	7 (36.84)	0.0267
Histological grades			
WDSCC	18 (58.06)	13 (41.93)	0.0358
MDSCC	10 (55.55)	8 (44.44)	0.5798
PDSCC	-	-	-

*FC* fold change.

### HOXA9 is significantly upregulated in OC at the protein level

The FFPE tissue blocks were further processed for Haematoxylin and Eosin (H&E) staining for histopathological analysis (Fig. 1L, upper panel). IHC analysis using HOXA9 antibody showed positive staining in the nucleus and cytoplasm of epithelial cells, whereas no staining pattern was observed in keratin pearls and connective tissues (Fig. 1L, lower panel). Upon image analysis, HOXA9 protein was significantly elevated in both PC and OC samples, particularly in 22 out of the 35 OC samples compared to normal samples (Fig. 1M). Additionally, a significant elevation of HOXA9 protein was observed in WDSCC and PDSCC (Fig. 1N). Persistent elevation of HOXA9 protein across progressive histological grades indicates its oncogenic role in OC. Further, association of HOXA9 protein expression with demographic and clinicopathological details of the patient samples were assessed (Supplementary Table 1).

### Knockdown of HOXA9 reduces the proliferation and colony growth of OC cells

**Fig. 2:**
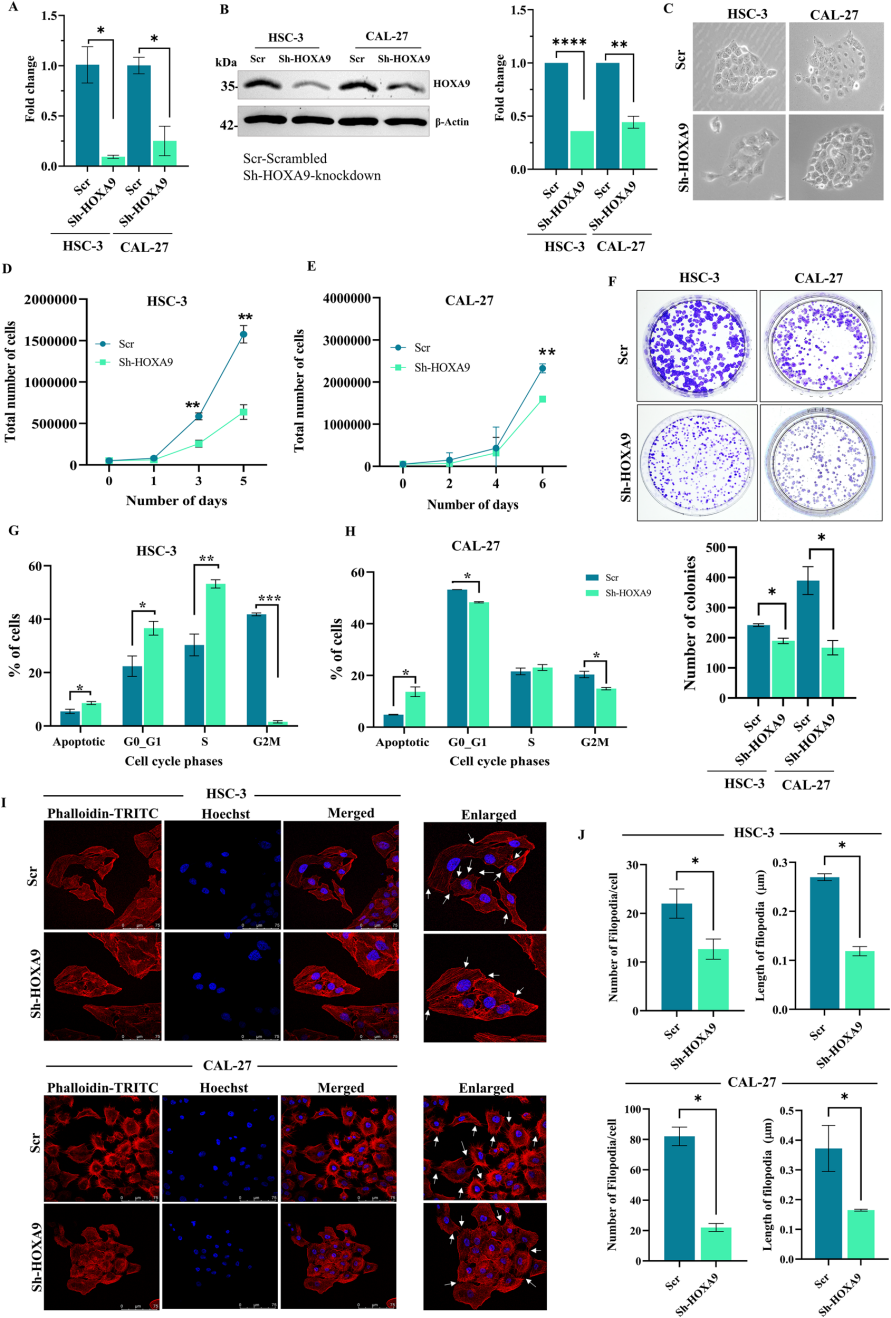
Effect of HOXA9-knockdown on cell proliferation, morphology, growth and cell cycle progression. **A** Bar plot showing the results of qRT-PCR showing the efficient knockdown of HOXA9 in HSC-3 and CAL-27 cells, at the mRNA level. **B** Representative western blot images confirming the knockdown of HOXA9 in HSC-3 and CAL-27 cells with β-actin as an endogenous control. Bar plot showing the results of quantitative densitometric analysis confirming the HOXA9-knockdown in HSC-3 and CAL-27 cells at protein level. **C** Bright field images showing the changes in the morphology of HSC-3 and CAL-27 cells upon successful HOXA9-knockdown (enlarged 10X magnification). **D** Line plots showing the gradual reduction in the proliferation rate upon HOXA9-knockdown with increase in the cell-doubling time of HSC-3 cells (32.68 h) and **E** in CAL-27 cells (46.90 h), compared to the corresponding scrambled cells (23.7 hours and 32.27 h), respectively. **F** Representative images of the anchorage-dependent colony formation assay (upper panel) and its quantitative analysis confirming the reduction in the number and size of the colonies upon HOXA9-knockdown in HSC-3 and CAL-27 cells, when compared to the scrambled cells (lower panel). **G** Cell cycle analysis of scrambled and HOXA9-knockdown cells using flow cytometry. Silencing of HOXA9 in HSC-3 cells showed a significant increase in the number of cells undergoing apoptosis, and G0/G1 cell cycle arrest (Scr v/s Sh-HOXA9: apoptotic phase 5.461% ± 0.80 v/s 8.58% ± 0.57; G0/G1 arrest 22.39% ± 3.80 v/s 36.60% ± 2.56). **H** In CAL-27 cells, the silencing of HOXA9 has significantly induced apoptosis (Scr v/s Sh-HOXA9: apoptotic phase 4.84% ± 0.12 v/s 13.70% ± 1.86), compared to the scrambled cells. **I** Confocal images of actin-phalloidin staining showing substantial changes in actin cytoskeletal rearrangements and the cell morphology upon HOXA9-knockdown in OC cells. The arrows indicate the presence of filopodia (63X magnification). **J** Box plots confirming the significant reduction in the number and length of filopodia upon HOXA9-knockdown in HSC-3 and CAL-27, respectively. Quantitatively, the number of filopodia in HSC-3 cells decreased from 22 ± 3 (mean ± SD) in scrambled (Scr) to 13 ± 2 in knockdown cells (Sh-HOXA9), and the length of the filopodia decreased from 0.26 µm±0.006 to 0.11 µm ± 0.009. In CAL-27 cells, the number of filopodia decreased from 82 ± 6 (mean ± SD) to 22 ± 2.64, and the length of the filopodia decreased from 0.37 µm ± 0.07 to 0.16 µm ± 0.003 following HOXA9 knockdown. The data represents mean ± SD of the experiments performed in duplicates, repeated twice and *P* < 0.05 was considered statistically significant.

Retroviral-mediated knockdown of HOXA9 was performed in HSC-3 and CAL-27 cells and confirmed at both mRNA and protein levels, respectively (Fig. 2A, B). Silencing HOXA9 induced changes in morphology of both OC cells (Fig. 2C). To understand its functional role in OC, we performed a series of functional assays. Notably, knockdown of HOXA9 reduced cell proliferation, as evidenced by an increased doubling time compared to scrambled cells (Fig. 2D, E). Furthermore, knockdown of HOXA9 significantly reduced the colony-forming ability of both OC cells (Fig. 2F), with a significant decrease in both number and size of the colonies.

### Knockdown of HOXA9 induces G0/G1 cell cycle arrest and apoptosis

Reduced proliferation was further confirmed via cell cycle analysis following HOXA9 knockdown. A notable increase in apoptosis (Supplementary Fig. 1), G0/G1 cell cycle arrest, and S-phase cells was observed upon HOXA9 silencing in HSC-3, likely reflecting a subpopulation of cells bypassing the G1 checkpoint and entering S-phase for faulty or aberrant replication (Fig. 2G). In contrast, knockdown of HOXA9 in CAL-27 cells drove a major population of cells towards apoptosis rather than cell cycle arrest, leading to the reduced number of cells undergoing subsequent G0/G1 and G2M arrest checkpoints (Fig. 2H).

### Knockdown of HOXA9 induces changes in size, shape, and morphology and actin-cytoskeletal rearrangement

Actin-phalloidin staining have revealed that knockdown of HOXA9 in HSC-3 and CAL-27 cells induced notable alterations in cell morphology, actin cytoskeletal organization, and increased cell size as indicated by increased cellular area and perimeter (Supplementary Fig. 2). Additionally, the knockdown cells showed enhanced cell‒cell adhesion and exhibited decreased number and length of filopodia (Fig. 2I, J).

### HOXA9 knockdown inhibits the migration and invasion of OC cells

**Fig. 3:**
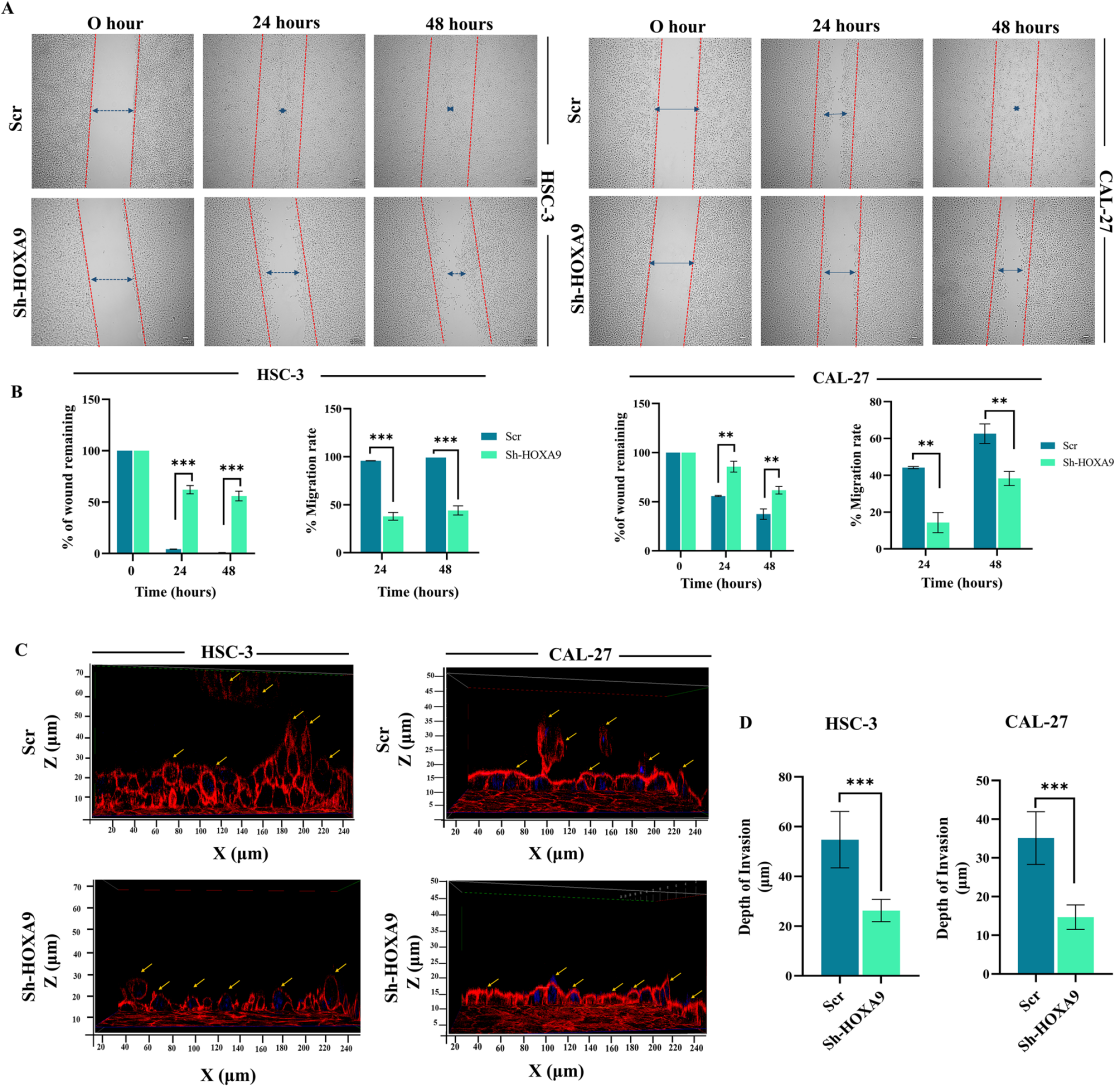
Effect of HOXA9-knockdown on the migration and invasion of OC cells. **A** Representative bright field image from the scratch assay demonstrating the reduction in the migration rate of HSC-3 and CAL-27 cells upon HOXA9 knockdown at 24 and 48-h intervals under 4X magnification. Notably, the wound was closed completely in scrambled OC cells by 48 h. **B** Box plots showing the quantitative estimation of the percentage of wound remaining and migration rate upon HOXA9-knockdown in HSC-3 cells. The migration rate in HOXA9-knockdown cells was significantly reduced at 24 h (37.88% v/s 95.74%) and 48 h (44.04% v/s 99.11%) compared to the scrambled HSC-3 cells. Similarly, the box plots for CAL-27 cells indicate a quantitative estimation of the percentage of wound remaining and migration rate upon HOXA9-knockdown in CAL-27 cells. The percentage migration rate in HOXA9-knockdown cells at 24 h (14.31% v/s 48.17%) and 48 h interval (38.27% v/s 62.59%) was significantly lower when compared to the scrambled CAL-27 cells. **C** Representative confocal Z-stacks images of 3D collagen gel invasion assay showing a significant reduction in the depth of invasion of HOXA9-knockdown HSC-3 and CAL-27 cells, when compared to the highest depth of invasion observed in corresponding scrambled cells. **D** Box plots showing the results of quantitative estimation of depth of invasion upon HOXA9-knockdown in HSC-3 and CAL-27 cells. The average depth of invasion was calculated by analyzing the images of Z-slices. The scrambled HSC-3 and CAL-27 cells showed a maximum depth of invasion at 54.71 ± 11.3 μm and 35.13 ± 6.82 μm, respectively, whereas knockdown cells showed comparatively lower depth of invasion i.e., 26.25 ± 4.49 μm in HSC-3 cells and 14.68 ± 3.17 μm in CAL-27 cells. The graphs represent mean ± SD of the experiments performed in duplicates, performed twice and *P* < 0.05 was considered statistically significant.

Upon knockdown of HOXA9, migratory ability of OC cells at 24 and 48 hours was significantly reduced, compared to scrambled cells (Fig. 3A). Image analysis revealed that percentage of wound remaining was significantly greater, and the migration rate was lower upon HOXA9 knockdown (Fig. 3B). Moreover, 3D collagen gel invasion assay demonstrated that HOXA9 silencing significantly inhibited the invasion (Fig. 3C, D), as evidenced by the reduced depth of invasion of OC cells.

### Knockdown of HOXA9 inhibits tumor growth and metastasis in vivo

**Fig. 4:**
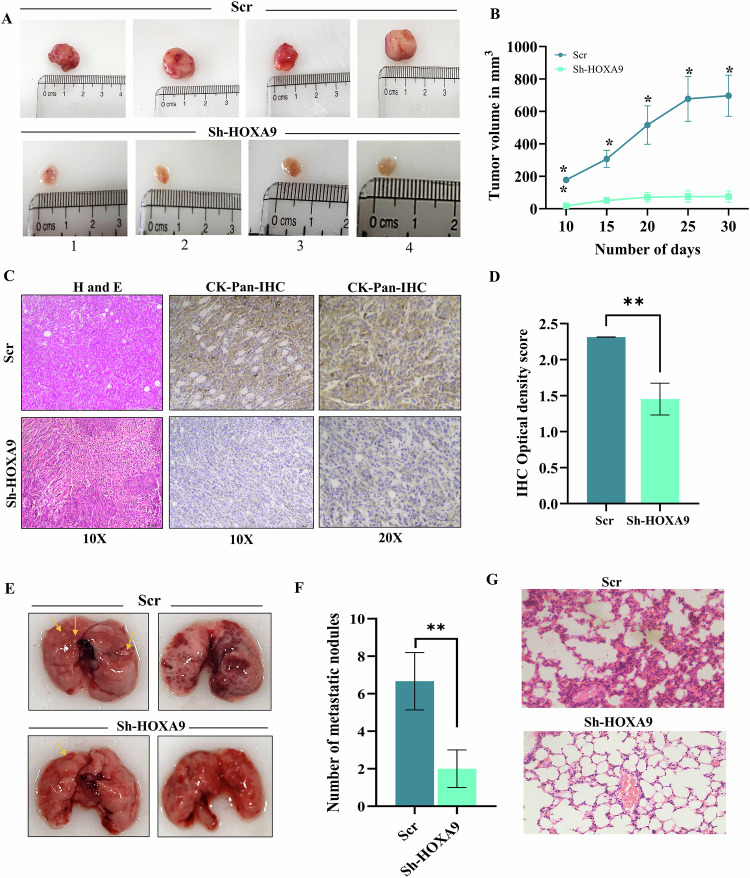
Knockdown of HOXA9 reduces tumor growth in vivo. **A** Representative images of tumors extracted from nude mice (*n* = 4). The mice that received scrambled HSC-3 cells showed progressively growing tumors. **B** The line graph represents the difference in the tumor volume of nude mice of scrambled and knockdown group as measured at regular time intervals. **C** Representative images of H&E staining and IHC staining for the tumor tissues stained with Pan-cytokeratin (CK-Pan) antibody. The tissues of scrambled group showing higher positive staining than that of knockdown group. **D** Bar graph showing the significant reduction in the IHC optical density score of the tissues of knockdown group mice when compared to scrambled group, stained with CK-Pan antibody. **E** Representative images of metastasized lung tissues in the presence and absence of HOXA9 expression. The arrows represent the metastatic nodules in mice lungs. **F** Bar graph showing the quantitative analysis of number of metastatic nodules in nude mice receiving scrambled and HOXA9-knockdown HSC-3 cells. **G** Representative images of H&E staining performed for mice lung tissues under 10X magnification. Mice that received scrambled cells exhibited extensive lung metastasis. *P* < 0.05 was considered statistically significant.

Nude mice studies were performed to assess the role of *HOXA9* in tumorigenesis. Scrambled cells formed tumors showing progressive growth, whereas mice administered with HOXA9-knockdown cells presented substantially smaller tumors (Fig. 4A, B). H&E staining of scrambled tumor showed an increased nucleus-to-cytoplasmic ratio, greater proportion of cells with abnormal nuclei, increased tumor cell density, increased pleomorphic and densely aggregated cells, compared to knockdown group (Fig. 4C). Notably, the optical density score of pancytokeratin stained xenograft tumor tissues was greater in the scrambled group, confirming the tissues of epithelial origin and successful tumor establishment and growth in the nude mice (Fig. 4C, D).

In the in vivo metastasis assay, the lung tissues were extracted following euthanasia, for subsequent processing (Fig. 4E). Systemic metastasis was observed in the lungs of the nude mice of scrambled group, with the greater number of metastatic nodules compared to knockdown group (Fig. 4F). H&E-stained images of lung tissues revealed denser and darker stained metastatic foci and metastatic nodules lacking an organized alveolar structure in scrambled group, compared to lighter and open structures of healthy lung tissues in knockdown group (Fig. 4G), indicating the intricate role of HOXA9 in OC metastasis.

### Inhibition of cancer-associated signaling pathways upon HOXA9 knockdown

**Fig. 5:**
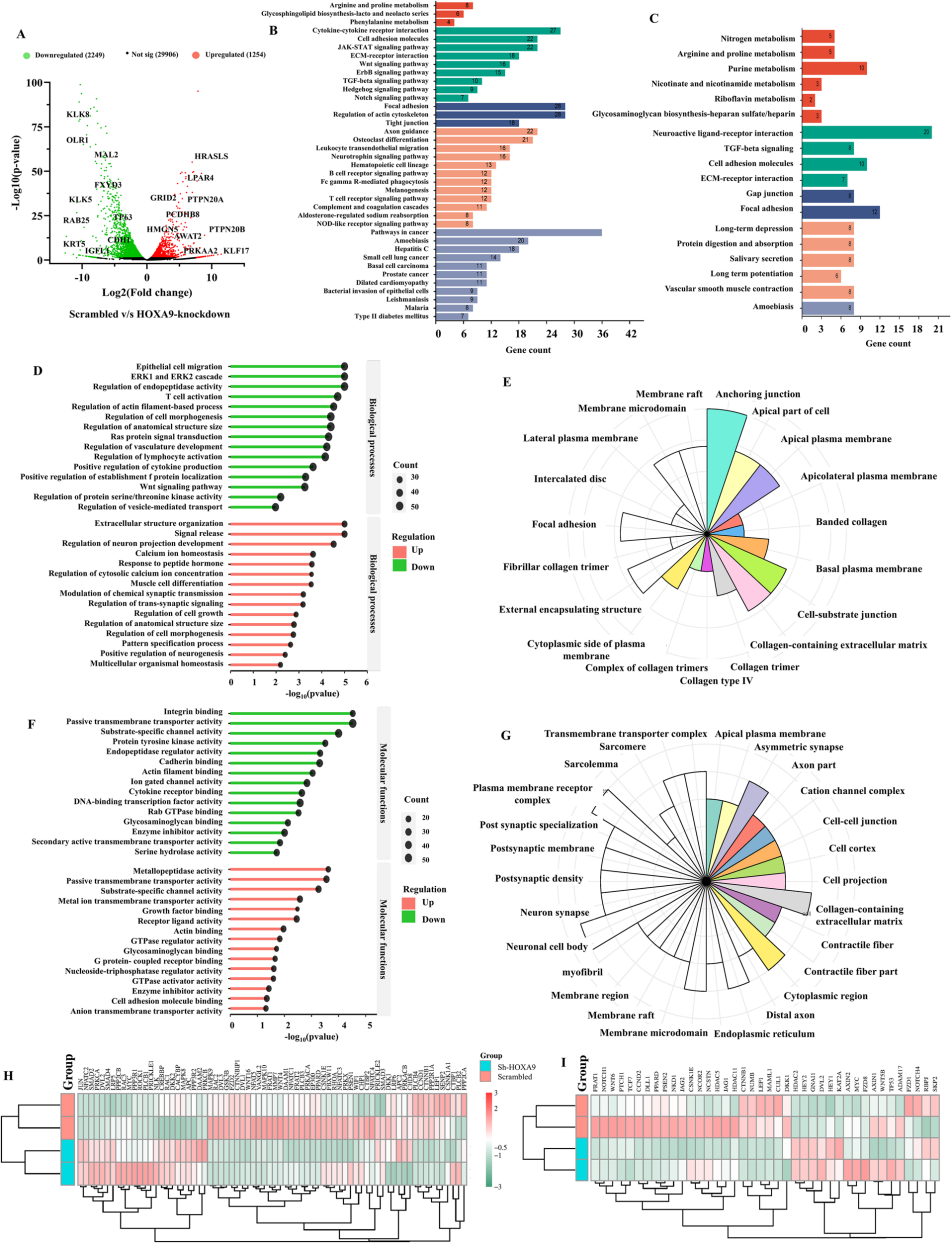
Differentially expressed genes and their signaling pathways upon HOXA9-knockdown. **A** Volcano plot representing the total number of downregulated and upregulated genes after HOXA9-silencing in HSC-3 cells, highlighting the top 10 upregulated and downregulated genes. **B** KEGG pathway analysis of downregulated and **C** upregulated genes. The knockdown of HOXA9 significantly reduced cancer-associated pathways and induced regular physiological functions. **D** Enrichment analysis of the biological processes associated with the downregulated genes and upregulated genes. **E** Cellular components associated with the downregulated genes. **F** Enrichment analysis of molecular functions of downregulated and upregulated genes. **G** Cellular components associated with the upregulated genes. **H** Heatmap showing the differential expression of Wnt pathway regulators upon HOXA9-knockdown. **I** Heatmap showing the differential expression of Wnt pathway target genes upon HOXA9-knockdown.

Differential gene expression analysis revealed that 2249 genes were downregulated, and 1254 genes were upregulated following HOXA9 knockdown (Fig. 5A). Moreover, KEGG pathway analysis of the differentially expressed genes (DEGs) identified 43 pathways that were downregulated in HOXA9-depleted cells, with the most significant being “pathways in cancer,” which included the downregulation of 42 genes. The cancer-related key genes including *ITGA2, WNT3A, TGFB2, WNT4, CSF1R, FN1, MAPK10*, *FGF3, IL6, LEF1, PTGS2* and *CBLC* were notably downregulated upon HOXA9 silencing, signifying the critical role of HOXA9 in OC progression. This study also revealed downregulation of JAK/STAT signaling, cytokine–cytokine receptor interactions, Wnt signaling, NOD-like receptor and Notch signaling and TGF-β signaling (Fig. 5B). Among the 19 upregulated pathways, “neuroactive ligand‒receptor interaction” was highly upregulated (including 20 upregulated genes). This upregulation could restore the cell‒cell communications, immune responses, and other physiological functions, leading to a shift toward normal cellular behavior. In addition, purine and riboflavin metabolism, protein digestion and absorption, ECM-receptor interaction, arginine and proline metabolism, and focal adhesion, were observed (Fig. 5C). These findings indicate that silencing of HOXA9 induces potential reversion to a more typical, less malignant cellular state.

### Knockdown of HOXA9 inhibits critical oncogenic processes in OC

The genes enriched in Ras and Rho protein signal transduction, Wnt signaling, ERK1 and ERK2 cascades, epithelial cell migration, growth, proliferation, angiogenesis, calcium ion transport, actin cytoskeleton organization, and stress-activated MAPK cascade were downregulated upon HOXA9-knockdown (Fig. 5D, upper panel). Most of these proteins were predominantly found in cytoplasm and leading edge of the cell (Fig. 5E). The downregulation of these biological processes (BP) was associated with molecular functions (MF) such as transcription factor activity, cytokine receptor binding, protein tyrosine kinase activity, Rab GTPase and Rho GTPase binding, voltage-gated ion channel activity, β-catenin binding, TGF-β receptor binding, and calcium-activated cation channel activity (Fig. 5F, upper panel).

Interestingly, HOXA9 depletion restored the physiological functions, especially signal release, extracellular structure organization, regulation of membrane potential, calcium ion homeostasis, regulation of cell size and anatomical structure, cell morphogenesis and pattern specification processes (Fig. 5D, lower panel) due to the induction of metallopeptidase activity, passive transmembrane transporter activity, GTPase activator activity, binding of cell adhesion molecules and activity of receptor-ligands (Fig. 5F, lower panel), which are associated in cytoplasm, cell‒cell junctions and receptor complexes (Fig. 5G). All the DEGs and their GO enrichment analysis results are provided in Supplementary Tables 2–6.

### HOXA9 regulates the Wnt/β-catenin signaling in OC

**Fig. 6:**
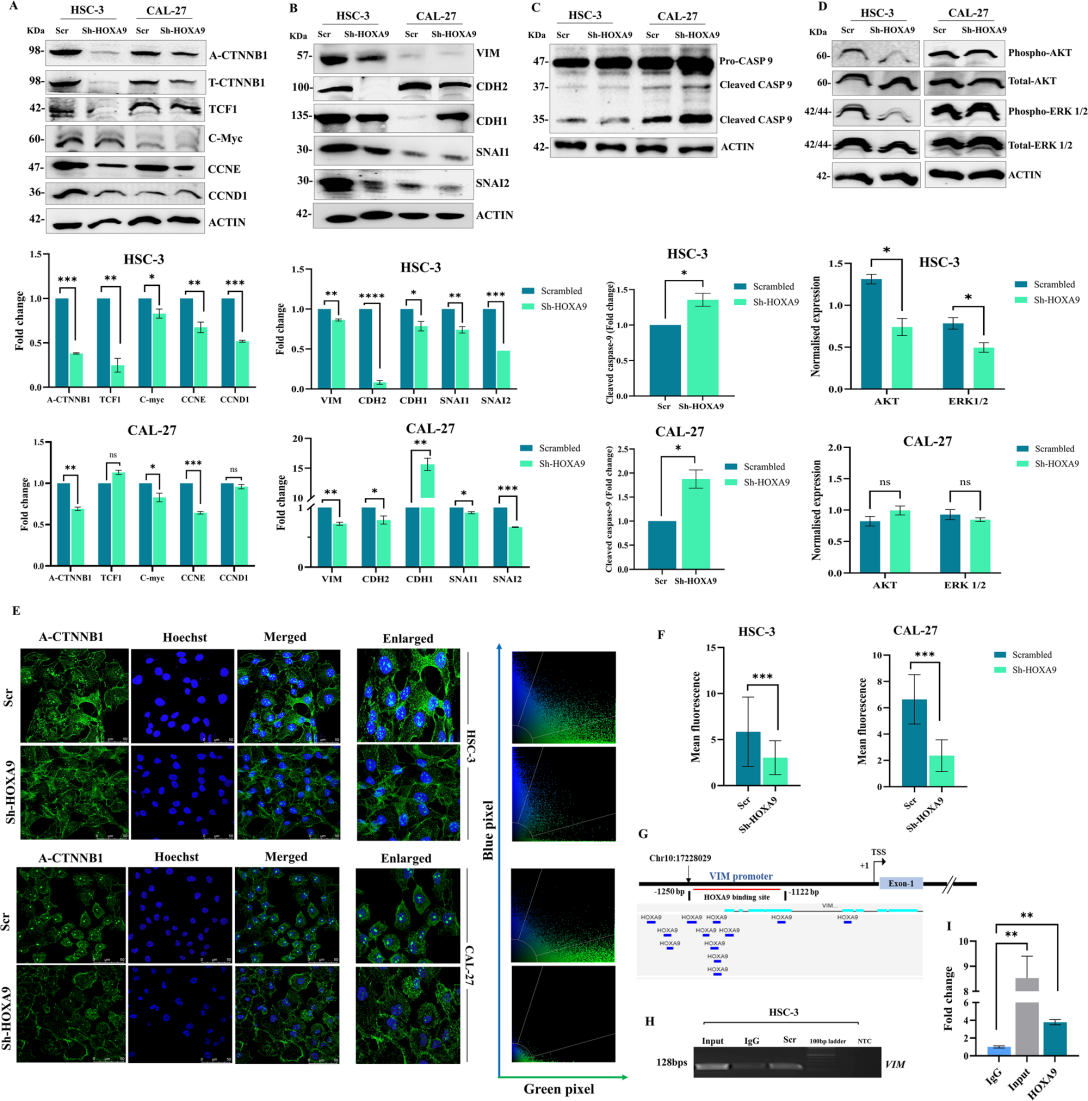
HOXA9-mediated regulation of signaling pathways in OC. **A** Western blot images and quantitative analysis (bar graphs) of proteins of Wnt/β-catenin pathway. Knockdown of HOXA9 leads to concomitant reduction in the protein levels of the members of Wnt pathway (A-CTNNB1, TCF1) and its targets (CCND1, CCNE, C-Myc) in HSC-3 and CAL-27 cells. **B** Western blot images with their quantitative analysis (bar graphs) of proteins of EMT pathway. Knockdown of HOXA9 significantly reduced the protein expression of mesenchymal markers (CDH2, VIM) and EMT transcription factors (SNAI1, SNAI2) with the simultaneous increase in the protein levels of CDH1. **C** Western blot images with their quantitative analysis (bar graphs) of apoptotic marker capsase-9 and its active form (cleaved caspase-9) showed significant upregulation of active form- cleaved caspase-9 in CAL-27 cells with no difference of its protein levels in HSC-3 cells. **D** Western blot images with their quantitative analysis (bar graphs) showing the reduction in phosphorylated ERK1/2 and AKT upon HOXA9 knockdown. **E** Representative confocal images showing nuclear accumulation of active-CTNNB1 in scrambled HSC-3 and CAL-27 cells and reduced nuclear translocation of active-CTNNB1 in HOXA9-knockdown cells. Mean florescence of active β-catenin in scrambled HSC-3 and CAL-27 cells was 5.84 ± 3.77 and 6.64 ± 1.87, whereas in HSC-3 and CAL-27 knockdown cells was 3.02 ± 1.84 and 2.357 ± 1.20, respectively. **F** Bar graphs represent quantitative analysis showing reduced accumulation of active-CTNNB1 upon HOXA9 knockdown. **G** Schematic representation of Vimentin (*VIM*) promoter showing the binding sites of HOXA9 transcription factor, predicted using computational databases. **H** Representative gel image of ChIP-PCR. **I** Quantitative analysis confirming the direct binding of HOXA9 transcription factor to *VIM* promoter (-1250 bp to −1122 bp upstream to TSS).

Despite identifying multiple pathways altered by HOXA9 in OC, we focused on Wnt/β-catenin pathway because of its close interaction with other oncogenic pathways that govern proliferation, migration, and EMT. DEGs were classified into Wnt pathway regulators and Wnt pathway targets using the Gene Set Enrichment Analysis (GSEA) web portal. Interestingly, a noteworthy reduction in levels of Wnt pathway regulators, namely, *WNT4, WNT16, CTNNB1, DVL1, DKK1*, and *GSK3B* and also downregulation of target genes, such as *CCND2, JAG1, JAG2, LEF1*, and *MYC*, were detected (Fig. 5H, I). Further, western blot analysis validated that HOXA9 depletion caused significant downregulation of Wnt pathway members, namely, active-CTNNB1, TCF1, C-Myc, CCNE, and CCND1 proteins (Fig. 6A). As evidenced by immunofluorescence staining, downregulation of HOXA9 was correlated with reduced nuclear accumulation of active β-catenin in OC cells (Fig. 6E, F). To our knowledge, this is the first study to demonstrate HOXA9-mediated nuclear translocation of active β-catenin in OC cells, leads to the activation of Wnt/β-catenin target genes.

### HOXA9 regulates epithelial‒mesenchymal transition in OC

Our RNA sequencing analysis revealed differential expression of key genes associated with EMT. To validate these findings and to investigate the role of HOXA9 in EMT, we performed western blotting which demonstrated that silencing of HOXA9 reduced the protein expression of VIM, CDH2, SNAI1, and SNAI2 in both OC cells. While HOXA9 knockdown concomitantly induced the expression of epithelial marker, CDH1 in CAL-27 (Fig. 6B), we observed a slight reduction in the CDH1 levels upon HOXA9 silencing in HSC-3 cells. This might be due to the invasive and mesenchymal characteristics of HSC-3 cells with strongly active EMT machinery, where other EMT transcription factors might dominate and induce alternative or compensatory pathways to suppress its expression despite HOXA9 knockdown [42, 43]. Furthermore, a marked increase in the levels of cleaved caspase-9 was observed following HOXA9-knockdown (Fig. 6C), indicating the activation of instrinic pathway of apoptosis upon EMT suppression. Additionally, knockdown of HOXA9 in HSC-3 cells inhibited the phosphorylation of AKT and ERK1/2 without altering their total protein levels (Fig. 6D).

### HOXA9 facilitates the transcriptional activation of VIM

Biological assays and western blot analysis demonstrated the crucial involvement of HOXA9 in EMT. To further validate these findings, we predicted the EMT-associated genes that are targeted by HOXA9 using in silico databases (Supplementary Fig. 3 and Supplementary Table 7). Among the EMT genes analyzed, HOXA9 transcription factor was predicted to bind to the *VIM* promoter from −1250 bp to −1122 bp upstream of the TSS (Fig. 6G), and ChIP‒PCR further confirmed this finding in HSC-3 cells (Fig. 6H, I).

### Knockdown of VIM impairs Wnt/β-catenin signaling in metastatic HSC-3 cells with elevated HOXA9 expression

To examine the functional consequences of HOXA9-mediated *VIM* activation on Wnt/β-catenin pathway, knockdown of VIM was performed in scrambled HSC-3 cells (Supplementary Fig. 4A) which naturally express high HOXA9 levels. VIM knockdown significantly reduced the WNT3A and DVL2 proteins, showed marked reduction in active-CTNNB1 levels and its downstream targets C-Myc and CCND1 (Supplementary Fig. 4B, C). This indicates that HOXA9-dependent upregulation of *VIM* extends beyond structural changes during EMT and is functionally associated with Wnt/β-catenin pathway in OC.

### Characterization of the functional promoters of HOXA9

**Fig. 7:**
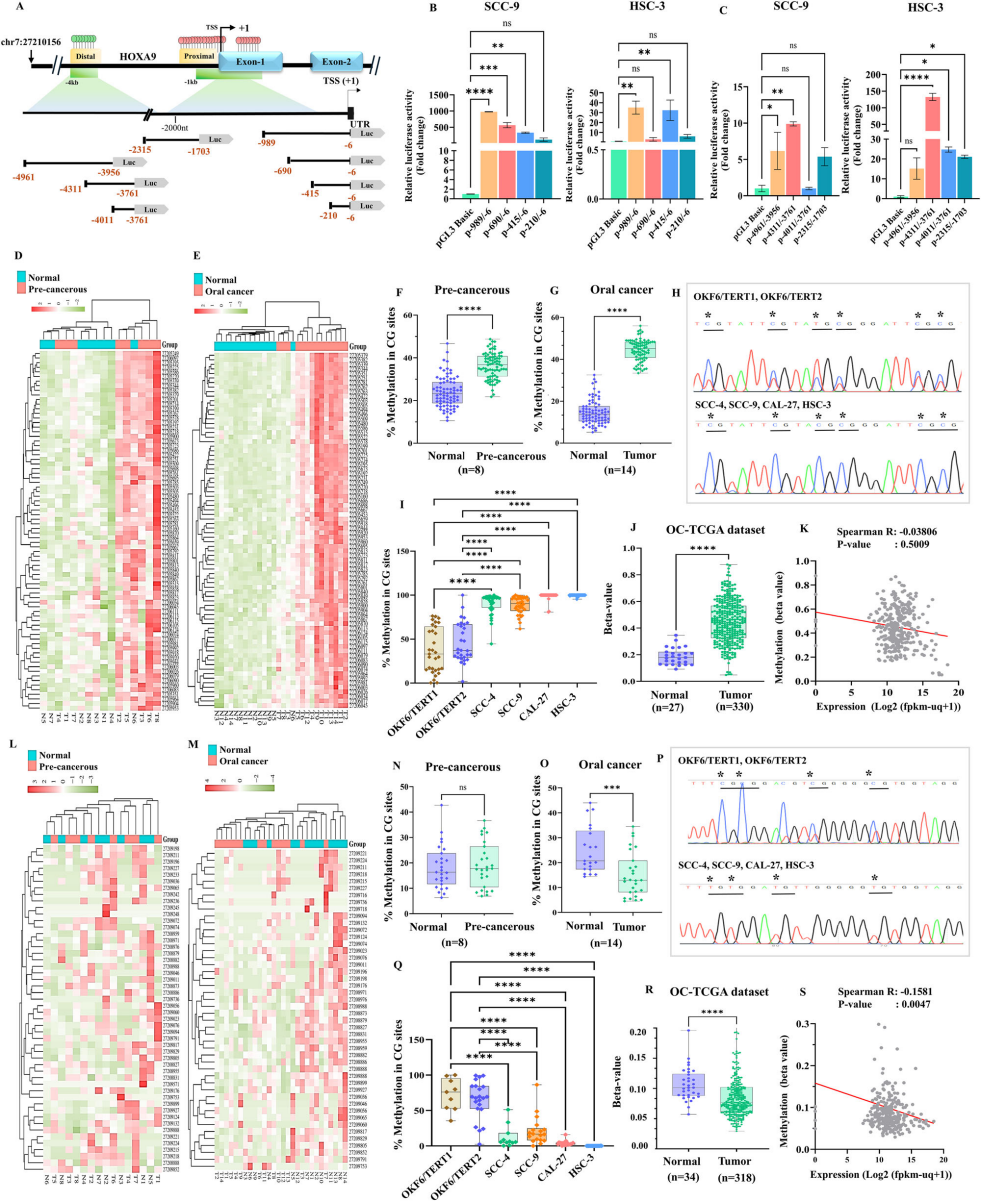
Promoter-DNA methylation analysis of HOXA9 in OC. **A** Genomic organization of *HOXA9* mapping to distal and proximal promoters, representing the regions used for luciferase assay. **B** Bar plots representing the relative luciferase activity of *HOXA9* proximal promoter construct pGL3-HOXA9 (−989 to −6bp) showing robust promoter activity in SCC-9 (FC: 977.5 ± 0.001), compared to HSC-3 (FC: 35.02 ± 0.0001). **C** Bar plots represent the relative luciferase activity of *HOXA9* distal promoter construct pGL3-HOXA9 (−4311 to −3761 bp) showing highest promoter activity in HSC-3 (FC: 132.42 ± 0.0009), compared to its activity in SCC-9 (FC: 9.89 ± 0.00004). Relative luciferase activity at negative control region pGL3-HOXA9 (−2315 and −1703 bp) showed lowest promoter activity in both SCC-9 (5.37 ± 1.24) and HSC-3 cells (21.09 ± 0.81). **D** Heat map representing the hierarchical clustering of methylation levels in the individual CG sites of proximal promoter with their chromosomal coordinates by Euclidean distance method in PC (*n* = 8) and **E** OC (*n* = 14) samples. **F** Box plots representing the MC-seq data showing the overall percent methylation in the individual CG sites of proximal promoter region in PC and **G** OC samples. Median difference of methylation PC v/s normal 12.37%, OC v/s normal 29.76%, *P* < 0.001. **H** Electropherogram representing the methylation pattern at *HOXA9* proximal promoter in cell lines. **I** Quantification of methylation of individual CG sites of *HOXA9* proximal promoter in cell lines. **J** The box plot representing the differential methylation at *HOXA9* proximal promoter in OC-TCGA dataset (beta-value difference 0.26, *P* < 0.0001). **K** Spearman correlation analysis between *HOXA9* expression and proximal promoter methylation of OC-TCGA dataset (Spearman R: −0.03, *P* = 0.50). **L** Heat map representing methylation in CG sites of distal promoter region plotted using same parameters as mentioned earlier, in PC (*n* = 8) and **M** OC (*n* = 14) samples. **N** Box plots representing the MC-seq data of *HOXA9* distal promoter in PC and **O** OC samples showing significant hypomethylation, compared to matched normal samples. Median difference of methylation OC v/s normal −12.65%, *P* < 0.001. **P** Electropherogram representing the methylation pattern at *HOXA9* distal promoter in cell lines. **Q** Quantification of methylation of individual CG sites of distal promoter showing significant hypomethylation in OC cells, compared to normal oral keratinocytes. **R** The box plot represents the differential methylation at *HOXA9* distal promoter in OC-TCGA dataset (beta-value difference −0.02, *P* < 0.0001). **S** Spearman correlation analysis between *HOXA9* expression and distal promoter methylation of samples of OC-TCGA dataset revealed significant negative correlation (Spearman R: −0.1581, *P* = 0.0047).

To investigate the regulation of *HOXA9* in OC, its promoter regions were cloned into luciferase reporter constructs, as schematically represented in Fig.7A. Dual luciferase reporter assay revealed a considerable increase in full-length proximal promoter (spanning -989bp to -6bp upstream to TSS) activity among the other deletion constructs in primary tumor derived- SCC-9 cells, compared to metastatic HSC-3 cells (Fig.7B). However, transient transfection of distal promoter region and other deletion constructs into OC cells, showed highest promoter activity at −4311 bp to −3761 bp region in metastatic HSC-3 cells, compared to SCC-9 (Fig. 7C), indicating the presence of enhancers or activators at distal promoter, making it a transcriptionally active region. Additionally, our findings revealed the increased activity of proximal promoter at non-metastatic stage and prominent elevation of distal promoter activity at metastatic stage, suggesting the stage-specific regulation of *HOXA9* transcription through differential promoter utilization.

### The HOXA9 proximal promoter is hypermethylated in OC samples

The heatmap generated from our methyl capture sequencing (MC-seq) analysis clearly indicates that *HOXA9* proximal promoter is highly methylated in PC and OC tissues, compared to matched normal (Fig.7D, E). which was further confirmed by quantitative analysis of individual CpG sites (Fig.7F, G). This finding was further validated through bisulfite Sanger sequencing of a 608 bp (spanning −497 bp to +111 bp) region containing 37 CpG sites. Average percentage of methylation in OC cells exceeded 80%, whereas it ranged from 25 to 50% in oral keratinocytes (Fig.7H, I). Furthermore, analysis of OC-TCGA dataset revealed a median β-value difference of 0.2658 between normal and primary tumor tissues (Fig.7J), with methylation in the primary tumor being the highest. However, Spearman’s correlation analysis did not reveal any significant correlation between *HOXA9* expression and methylation (Fig.7K), indicating the involvement of other factors in *HOXA9* regulation.

### The HOXA9 distal promoter is hypomethylated in OC samples

The differential methylation at each of the CG sites at distal promoter in PC and OC samples is represented in the form of a heatmap (Fig. 7L, M). MC-seq data analysis for the 550 bp region revealed no differential methylation in PC samples (Fig. 7N). Interestingly, we detected hypomethylation at distal promoter in OC samples compared with matched normal samples (Fig. 7O). This finding was further validated through bisulfite Sanger sequencing of a 628 bp region (spanning −4201 bp to −3574 bp) containing 48 CpG sites. Average percent methylation in OC cell lines was significantly low (12–40%), whereas greater methylation (65%) was observed in oral keratinocytes (Fig. 7P, Q). Notably, analysis of distal promoter methylation in OC-TCGA dataset showed a significant median β-value difference of -0.0245 between normal and primary tumor tissues (Fig. 7R). Spearman correlation analysis revealed a significant negative correlation between expression and methylation (Fig. 7S).

### Hypomethylated distal promoter enriched with activating histone marks drives HOXA9 upregulation

To examine whether DNA hypomethylation synergizes with histone modifications to regulate *HOXA9* in OC, we analyzed publicly available ChIP-seq datasets. Our analysis revealed the enrichment of H3K27Ac on the distal promoter in OC cells, including HSC-4, YD-8, BHY, CAL-33, BICR31, and BICR16 (Supplementary Fig. 5) and deposition of H3K4Me3 was observed on the distal promoter in normal cell lines (Data unavailable for OC cells). Collectively, these findings indicate that activating histone marks enriched on the hypomethylated distal promoter might be involved in the upregulation of *HOXA9* in OC, requiring further validation. Hence, on the basis of the methylation changes detected in the patient’s DNA, hypomethylation at the distal promoter could serve as a clinical marker for monitoring cancer progression to advanced stages.

## Discussion

Studies have depicted the dual nature of *HOXA9* as a tumor promoter and tumor suppressor in different malignancies. Downregulation of *HOXA9* in different gynecological cancers [21, 22, 44, 45], lung cancers [46–48], and skin cancers [26, 27, 49] has been significantly associated with invasion, metastasis, and a recurrent phenotype. However, in PCa [41], Osteosarcoma (OS) [50], and gastro-intestinal cancers [23, 51–54], *HOXA9* levels were considerably elevated and linked to positive lymph node metastasis and TNM staging. The present study revealed the overexpression of *HOXA9* in the advanced stage of OC, which was associated with lymph-node metastasis and higher histological grade. Notably, this validation across a broader cohort of patient samples further substantiated its clinical relevance in OC. This observation was consistent with the elevation of *HOXA9* in HNSC subtypes, especially in nasopharyngeal carcinoma (NPC) [55]. and laryngeal carcinoma (LC) [56].

Understanding its intricate role in OC progression and metastasis is crucial for targeting HOXA9 therapeutically. The functional studies confirmed that retroviral-mediated silencing of HOXA9 in OC cells significantly reduced proliferation, growth, migration, and invasion; initiated apoptosis in cell models in vitro; and markedly decreased tumor growth and metastasis in in vivo nude mice models. Researchers have shown that the induction of proliferation, migration, invasion, and metastasis is predominantly driven by the Wnt/β-catenin cascade in several cancers [57]. while also triggering the genes involved in EMT [58]. Our study revealed that HOXA9 favors the aggressive behavior of tumor mainly by regulating two crucial events-EMT and metastasis. To validate these findings, we present novel evidence that i) HOXA9 inhibition in OC models caused a substantial reduction in tumor aggressiveness, EMT, and metastasis, with a concomitant reduction in nuclear translocation of active β-catenin, subsequently causing a reduction in the levels of Wnt target genes (C-Myc and CCND1), along with increased expression of CASP9. ii) HOXA9 also functions as a potential inducer of EMT by the transcriptionally activating *VIM* and its depletion further reduced expression of EMT transcription factors, including SNAI1 and SNAI2. Past studies have shown that HOXA9 acts as a transcriptional activator of crucial signaling molecules in cancer, especially those involved in EMT [21, 22, 24–27, 44]. Herein, we demonstrated the binding of transcription factor HOXA9 to the *VIM* promoter at -1250bp to -1122bp upstream of its TSS. Consistent with previous findings, we observed a reduced p-ERK1/2 and EMT-TFs with diminished A-CTNNB1 upon HOXA9 knockdown [59]. Moreover, EMT is a multidimensional phenomenon for invasion and metastasis, which can be induced in both β-catenin-dependent and β-catenin-independent manner [58, 60]. Studies highlight the significance of Wnt/β-catenin/EMT axis in metastasis and therapy resistance in various cancers [58]. Towards this, knockdown of *VIM* in *HOXA9* overexpressing OC cells showed a substantial reduction in the Wnt3A ligands and active β-catenin levels, leading to reduced expression of target genes (C-Myc and CCND1). Thus, our study unveiled a functional link between *VIM* and Wnt/β-catenin signaling, where HOXA9-dependent transcriptional activation of *VIM* cooperates with Wnt/β-catenin cascade to promote EMT and OC metastasis.

Consistent elevation of *HOXA9* in OC underscores the necessity of investigating the epigenetic determinants governing its activation. Studies have reported the ncRNA-mediated epigenetic regulation of *HOXA9* and its clinical importance in OC [36, 61, 62]. While exploring the promoter-DNA methylation, researchers reported that hypermethylation of *HOXA9* promoter in the salivary rinses and tissues of patients could represent a reliable biomarker for OC diagnosis [31, 38]. Similarly, our data from high-throughput MC-Seq and analysis of the OC-TCGA dataset demonstrated the proximal promoter hypermethylation. Despite evidence indicating *HOXA9* hypermethylation in OC, no studies have demonstrated its association with gene upregulation. To address this apparent contradiction, we further investigated the presence of distal *HOXA9* promoters. Computational analysis and validation with in vitro experiments, we found that transcriptionally active *HOXA9* promoter exhibited significant hypomethylation (located ~-4kb upstream of TSS) in OC tissues which is associated with *HOXA9* upregulation. Correlating *HOXA9* distal promoter methylation with its expression in OC-TCGA dataset showed a weak but statistically significant inverse correlation, indicating that *HOXA9* might be regulated via promoter DNA hypomethylation, but not the sole determinant of gene expression. Notably, hypomethylated promoter enriched with histone modifications facilitates oncogene activation in cancer [39, 40]. In PCa, researchers have reported that *HOXA9* overexpression is driven by TWIST1/WDR5-mediated H3K4Me3 deposition on its promoter [41]. Similarly, our study demonstrated that hypomethylation of *HOXA9* distal promoter enriched with H3K27Ac and H3K4Me3 activating histone marks, collectively establishes a transcriptionally active chromatin state to induce *HOXA9* upregulation in OC [40, 41, 63, 64]. Indeed, site-specific deletion or CRISPR-mediated knockout of the distal promoter in future studies will uncover this complexity of the epigenetic regulation of *HOXA9* in OC.

Collectively, the distinct pattern of *HOXA9* expression in diverse clinical contexts highlights its potential diagnostic and prognostic utility for guiding therapeutic strategies in OC. Our study provides compelling evidence that *HOXA9*, driven by a complex epigenetic mechanism that drives EMT and metastasis in OC by modulating the HOXA9/VIM/Wnt–β-catenin signaling axis. Silencing of HOXA9 significantly disrupts oncogenic pathways and impairs cancer-associated biological processes. Together, these findings provide a strong rationale for further investigations into HOXA9-targeted therapies to improve patient overall survival.

## Materials and methods

### Clinical specimen collection and assessment of the demographic and clinical characteristics of the study participants

A total of 75 clinical samples (along with 75 matched normal samples) were collected from patients upon their consent and ethical approval (IEC: 348/2018) from Kasturba Hospital, Manipal. Patients who underwent prior treatment and were HPV-positive were excluded from the study. Considering an effect size of 0.5 and 80% statistical power, with a Bonneferoni adjusted level of significance of 5% for multiple comparisons, a minimum of 25 samples per group were considered. Approximately 25 pre-cancerous (PC) samples along with 50 OC tissues with matched normal samples, were collected and divided into two groups: node-negative (NN) and node-positive (NP). PC samples comprise of oral lesions with dysplastic changes. Whereas NN group comprises TNM stage I and II in which cancer does not involve adjacent lymph nodes and NP group comprises stage III, IVa, and IVb in which cancer cells have been extended to adjacent lymph nodes. All the patients were clinically staged and histologically classified according to the WHO criteria. The demographic and clinical features of study participants are summarized in Supplementary Table 8.

### Cell line maintenance

The OC cell lines namely, SCC-4 and SCC-9 acquired from ATCC (Manassas, USA) were maintained in 1:1 ratio of DMEM: Ham’s F12 (HiMedia, India) with FBS (10%, HiMedia, India) supplemented with hydrocortisone (400 ng/mL, Sigma Aldrich, USA). Whereas CAL-27 and HSC-3 cells (ATCC, Manassas, USA) were cultured in DMEM media (HiMedia, India) with FBS (10%). Normal oral keratinocytes, OKF6/TERT-1 and OKF6/TERT-2 were grown initially in K-sfm (Thermo Fisher Scientific, USA). Once they reached one-third confluency, it was replaced with 1:1 (v/v) ratio of K-sfm: DF-K media (Thermo Fisher Scientific, USA) as described previously [65]. Cells were cultured in CO_2_ (5%) incubator at 37 °C and 95% humidity. All the cell lines were authenticated by STR profiling (Promega, USA).

### Animal models

Male athymic BALB/c nude mice aged approximately 5-6 weeks and weighing approximately 20–25 g were used for the study (*n* = 4/group) upon approval from the Animal Ethics Committee (IAEC/KMC/49/2018), Manipal. The animals were maintained under SPF conditions at a temperature of 20–25 °C, 45–60% humidity, and a light/dark cycle (12-h). The animals were handled aseptically under laminar airflow in an SPF facility. The experimental animals were kept in cages (four/cage) and provided with ad libitum food and sterilized water. Animal handling was performed as per the guidelines for the use and care of live animals by the US National Institutes of Health (NIH).

### RNA sequencing of OC tissue samples

Total RNA was isolated from PC (*n* = 7), NN (*n* = 6) and NP (*n* = 9) samples, along with matched normal samples using the mirVana™ Isolation Kit (Invitrogen, USA). The samples that passed the quality check were used to prepare the libraries using Ultra II Directional RNA-Seq Library Prep Kit (NEB, USA). The removal of rRNAs, purification and fragmentation followed by first-strand cDNA synthesis were performed using reverse transcriptase. Upon second-strand cDNA synthesis, the libraries were further processed to generate 60 M, paired-end reads (150 bp) using Illumina HiSeqX/NovaSeq 6000 instrument. Using the DESeq2 R package, raw reads were normalized and FPKM values were used to determine the differential gene expression in OC samples compared with the matched normal samples. Further information on the samples analyzed in the present study is summarized in Supplementary Table 9.

### Gene expression analysis with qRT‒PCR

Total-RNA isolation from cells and OC patient samples (*n* = 75, along with matched normal samples) was performed using the mirVana™ miRNA Isolation Kit (Invitrogen, USA). Isolated RNA (1 μg) was subjected to cDNA synthesis using a High-Capacity cDNA Reverse Transcription Kit (Applied Biosystems, USA) following the manufacturer’s instructions. TaqMan Gene Expression Assays for *HOXA9* (Hs04931837_mH) and endogenous control *ACTB* (Hs01060665_g1) (Invitrogen, USA) were performed using TaqMan™ Universal Master Mix (Applied Biosystems, USA). Approximately 100 ng of cDNA was used as a template for qPCR analysis, performed using QuantStudio 6 Pro (Thermo Fisher Scientific, USA). The relative expression was estimated following the Livak method [66].

### Generation of HOXA9 stable knockdown cell lines

To generate a stable knockdown cell line, HOXA9 shRNA retroviral plasmids (pGFP-V-RS) were purchased from Origene Technologies (TG307647) and transfected into Phoenix packaging cells using Lipofectamine 3000 reagent (Thermo Fisher Scientific, USA). Following the retroviral-mediated stable transduction protocol, efficient knockdown was performed in HSC-3 and CAL-27, in which *HOXA9* expression was high compared to normal oral keratinocytes. After 48 h of transduction, the cells were treated with 3 μg/mL (HSC-3) or 0.5 μg/mL (CAL-27) puromycin (Sigma Aldrich, USA) for 15–21 days to select positive clones. The extent of knockdown was confirmed by performing qRT‒PCR and western blotting. Here onwards, empty vector transduced cells are referred as “Scrambled (Scr)” and the cells transduced with HOXA9 shRNA, are referred to as “HOXA9-knockdown (Sh-HOXA9)”.

Additional methodology followed to perform experiments using clinical samples and cell lines, including immunohistochemistry (IHC), in vitro functional assays, immunofluorescence staining, Chromatin immunoprecipitation (ChIP), PCR, and methylation analysis have been provided as the “Supplementary materials and methods”.

### In vivo tumorigenicity assay

Scrambled and HOXA9-knockdown HSC-3 cells (3.5 × 10^6^cells/mL) were suspended in Matrigel (Corning, USA) at 1:1 ratio and subcutaneously injected (*n* = 4 per group) into the lower flanks of the animals. The tumor dimensions were analyzed externally at regular intervals via callipers, and the volume of the tumor was determined using the standard formula *V* = ab^2^/2 (a, length; b, width). As per the ethical guidelines, tumors were allowed to grow without exceeding the maximum dimensions required for the study. Thirty days after injection, the animals were sacrificed, the tumor tissues were removed, images were taken, the samples were fixed in buffered formalin, and the sections were further processed to prepare FFPE tissue blocks.

### In vivo metastasis assay

Approximately 1 × 10^5^ scrambled or HOXA9-knockdown cells were suspended in 0.1 mL PBS and administered via the tail vein of 6–8-week-old nude male mice for in vivo metastasis assays (*n* = 4/group). The animals were monitored for 30 days, and at the 5th week, they were euthanized, organs were collected, and the metastatic nodules in the lungs were quantified. Excised tissues were fixed in buffered formalin and further processed to prepare FFPE tissue blocks.

### H&E staining and immunohistochemistry (IHC)

Excised mice tissues were processed; H&E staining was performed as described earlier. IHC was performed on the tissues of the xenograft tumors by taking 4 μm sections, followed by staining with a Cytokeratin Pan Monoclonal antibody (1:1000, MA5-12231, Thermo Fisher Scientific, USA). Capturing and image processing were performed as described earlier (Supplementary materials and methods).

### Total RNA sequencing

Total RNA was extracted from scrambled and HOXA9-knockdown cells and subjected to RNA sequencing using the Illumina platform as discussed earlier. We followed the NEB Next RNA Ultra II directional protocol for library preparation, and approximately 60 million reads per sample (2 × 150 bp reads) were generated using Illumina HiSeq4000/X Ten/Novaseq platform.

### Bioinformatic analysis—Differential gene expression and pathway enrichment analysis

Once the raw data were generated, quality control and adapter trimming were performed using Trimmomatic, and contamination of the tRNA/rRNA sequences was removed using Bowtie2. These data were aligned with the hg19 assembly using HiSAT2, the raw reads were derived from FeatureCounts, and differentially expressed genes were identified using DESeq2 (R package). Analysis including, ORA and GSEA were performed using the clusterProfiler R package. The genes with a cut-off of ±1 and *P* ≤ 0.05 were considered for further analysis. To identify the pathways altered by the HOXA9 upon its knockdown, the genes showing significant differential expression were analyzed for KEGG pathway, gene ontology, and disease enrichment analyses.

### Western blot analysis

Scrambled and HOXA9-knockdown cells were homogenized in RIPA buffer, and the total proteins were resolved by electrophoresis on a 10% SDS‒polyacrylamide gel and then transferred on a nitrocellulose membrane (Bio-Rad, USA). The blotted membranes were blocked with BSA (5%) and incubated with primary antibodies, namely, HOXA9 (1:1000, sc-81291, Santacruz, USA), CTNNB1 (8480T), nonphospho-CTNNB1 (8814S), SNAI2 (9585T), C-Myc (5605 T), TCF-1 (2203T), CASP9 (9502T), CDH1 (3195 T), ACTB (4967S), phospho-AKT (9271S), Total-AKT (9272), phospho-ERK1/2 (4370S), Total-ERK1/2 (4695S, Cell Signaling Technology (CST), USA), CCND1 (ab134175, Abcam, UK), CCNE (sc-247, Santacruz, USA), CDH2 (PAB481Hu01), SNAI1 (PAK089Hu01), and VIM (1:3000, PAB040Hu01, Cloud Clone, USA), at 4 °C overnight and next day with corresponding secondary antibodies anti-mouse (1:5000, sc-516102, Santa Cruz, USA), or anti-rabbit (7074P2, CST, USA) for 2 h. Bands were visualized with Clarity Western ECL Substrate (Bio-Rad, USA) and captured with an Image Quant LAS 4000 (GE Healthcare, USA) or iBright 1500 (Invitrogen, USA). The band intensities were quantified using ImageJ software.

### Knockdown of VIM in HSC-3 cells and western blot analysis of Wnt/β-catenin signaling markers

Transient knockdown of VIM in scrambled HSC-3 cells was performed using predesigned, validated shRNA construct (TRCN0000029121, Merck, Bangalore) using Lipofectamine 3000 reagent (Thermo Fisher Scientific, USA). After 48 h of transfection, the cells were treated with 3 μg/mL puromycin (Sigma Aldrich, USA) for 15–21 days to select positive clones. The extent of knockdown was confirmed by performing western blotting using VIM (1:3000, PAB040Hu01, Cloud Clone, USA) antibody as described previously. Western blotting was performed for the markers of Wnt/β-catenin pathway namely, WNT3A (2721T), nonphospho-CTNNB1 (8814S), CTNNB1 (8480T), DVL2 (3224T), TCF1 (2203T), C-Myc (5605 T, CST, USA), CCNE (sc-247, Santacruz, USA) and CCND1 (ab134175, Abcam, UK), with ACTB (4967S, CST, USA) as endogenous control, as described previously.

### Cloning the promoter regions, dual-luciferase reporter assay, and promoter validation

Different computational tools, such as UCSC genome browser [67]. EPDnew [68]. and PROMO-alggen [69]. which provides the high-throughput CAGE and Oligocapping data of eukaryotic Pol II promoters where TSS has been determined experimentally. For the conceptual clarity, we have designated the region located −1 kb upstream of the TSS as “proximal” promoter and the region located ~−4 kb upstream to TSS was designated as “distal” promoter. These promoters and their corresponding deletion constructs were cloned separately into KpnI and NheI/HindIII (NEB, USA) restriction sites of pGL3 basic vector (Promega, USA), confirmed by restriction digestion and DNA sequencing. List of the primers used for cloning are available in Supplementary Table 10 and the list of promoter constructs are available in Supplementary Table 11.

Approximately 1 × 10^5^ cells were seeded in 12-well plates, and the plasmids were individually cotransfected with the pRL-SV40 vector using Lipofectamine 3000 (Thermo Fisher Scientific, USA). After 48 h post transfection, the cells were lysed, and reporter activity was measured with dual luciferase reporter assay kit (Promega, USA) using FB12 Luminometer (Berthold Technologies, Germany) The luciferase readings (RLU/s) were normalized to readings of Renilla, and the results are presented as the fold changes relative to the empty pGL3 basic vector.

### Methyl capture sequencing (MC seq)

The genomic DNA from PC (*n* = 8), NN (*n* = 6), and NP (*n* = 8) OC tissue samples was isolated using QIAamp Fast DNA Tissue Kit, Qiagen. Quality checks, genomic library construction, adapter ligation, purification and hybridization were performed according to the Illumina-compatible SureSelectXT Methyl-Seq Target Enrichment System protocol (llumina Multiplexed Sequencing, Agilent Technologies, USA) following the manufacturer’s instructions. Magnetic Dynabeads (Invitrogen, Carlsbad, CA, USA) were used to capture hybridized library fragments. Using captured library, bisulfite conversion (EZ DNA Methylation Gold Kit, Zymo Research, USA) followed by PCR amplification was performed. Paired-end sequencing (150 cycles) was performed using Illumina HiSeq X Ten sequencer (Illumina, USA), and the Illumina reads were quality checked using FastQC v0.11.3 [70]. and low-quality reads (score <30) and short reads (<20 bp) were removed using TrimGalore v0.4.0 (Babraham Bioinformatics, UK). We have used Bismark for aligning high-quality reads to hg19 human reference genome due to compatibility with probe annotations and alignment workflows. The frequencies of C and T in each of the CG sites extracted after processing were used for further differential methylation analysis for computing the variance between the OC and matched normal samples. The differences in the methylation levels were visualized via a heatmap.

### Retrieval of publicly available ChIP seq datasets and visualization

To determine the enrichment of histone modifications on the distal promoter of *HOXA9* in OC, we have explored publicly available ChIP-seq data using Cistrome DB database [71]. This database provides ChIP-seq, DNase-seq and ATAC-seq chromatin profiling data which provide the histone post-translational modifications at desired chromosomal locations (hg38 assembly) and transcription factor binding sites, curated and analyzed from multiple sources such as ENCODE and Epigenomics road map project. Referring to this database, we have retrieved the histone modifications data for *HOXA9* distal promoter region (1200 bp region, validated in our study) in OC cell lines and visualized it in UCSC genome browser (Human-GRCh38/hg38).

### Statistical analysis

All the statistical tests, namely, Student’s t test (paired or unpaired), ANOVA (one-way or two-way), and multiple t tests, were carried out using the GraphPad Prism (free version-online). Parametric or non-parametric tests were performed based on normality of data distribution. Data were presented as mean ± SD and the data with *P* ≤ 0.05 regarded as statistically significant. All the experiments were performed in duplicates and repeated twice.

#### Ethical approval

For human studies, approval was taken from Institutional Ethics Committee of (IEC: 348/2018), of the Kasturba Hospital, Manipal. For animal studies, approval was taken from the Institutional Animal Ethics Committee (IAEC/KMC/49/2018), Manipal.

## Supplementary information

Supplementary materials and methods

Supplementary Tables

Supplementary Figures

Uncropped Western blot

## Data Availability

The RNA-sequencing and methyl capture sequencing (MC-seq) raw data generated from clinical tissue samples in this study have been deposited in NCBI SRA (Sequence Read Archive) with Bio project accession numbers PRJNA1357308 and PRJNA1366722, respectively. Additionally, uncropped western blot images from the present study are provided with this manuscript. Other original data and their protocol can be available from the corresponding author upon reasonable request.
