# Supplementary material for: HOXA9 orchestrates EMT and metastasis in oral cancer via transcriptional activation of vimentin and β-catenin signaling
Source: Cell Death Dis. 2026 Mar 28;17(1):428. doi: 10.1038/s41419-026-08664-7 (PMC13153177; doi:10.1038/s41419-026-08664-7)
Supplement: Supplementary file 1 — Supplementary materials and methods [file 41419_2026_8664_MOESM1_ESM.doc]

**HOXA9 orchestrates EMT and metastasis in Oral cancer via transcriptional activation of vimentin and β-catenin signaling**

**SUPPLEMENTARY *-*Materials and Methods:**

***Clinical significance of HOXA9:***

The collected clinical samples were stratified according to tumor site, clinical stage, and histological grade according to the standard TNM staging and grading system. This stratification was crucial for understanding the clinical relevance of *HOXA9* expression. We performed qRT‒PCR to assess the expression of *HOXA9* in different tumor sites, including the buccal mucosa, gingivo buccal sulcus, tongue, alveolus, maxilla, and floor of the mouth. Additionally, *HOXA9* expression was evaluated in various clinical stages (stages I, II, III and IV) and across varying histological grades, namely, well-differentiated (WDSCC), moderately differentiated (MDSCC), and poorly differentiated squamous cell carcinoma (PDSCC). This comprehensive analysis was essential for understanding the correlation of *HOXA9* expression with tumor progression, aiding in the identification of its potential role as a biomarker for prognosis and therapeutic targeting in OC.

***Cross-validation of gene expression using public datasets:***

A cross-validation study was performed using the UCSC Xena browser (1). By retrieving the GDC-TCGA HNSC gene expression dataset, we stratified the samples based on the tumor site and thus analyzed the OC-TCGA data (normal (n=19) and tumor samples (n=247)) using GraphPad Prism (free online version). Furthermore, data from GEO datasets with accession IDs GSE23558 (2) and GSE37991(3), reposited in NCBI database, were retrieved and analyzed, which included OC samples along with samples collected from normal healthy individuals.

***Immunohistochemistry (IHC) and image analysis:***

Normal (n=16), PC (n=5), and OC (n=35) samples were further processed to prepare FFPE tissue blocks. Approximately 5μm sections were taken and H&E staining was performed using standard protocol. Images of the stained slides were captured using a brightfield microscope (Olympus, Japan). which was then subjected to histopathological examination by two independent clinicians. Approximately 4μm sections were taken on APES (3-Aminopropyltriethoxysilane) coated slides. The tissues were deparaffinized and subjected to antigen retrieval at 110° C for 10 minutes using Tris-EDTA buffer with pH 9.0. The activity of endogenous peroxidase was blocked with H_2_O_2_ (3%). The sections with activated antigens were subsequently incubated overnight with an anti-HOXA9 antibody (1:100, Santa Cruz, USA) at 4°C moist chamber. The tissue sections were then treated following the PolyExcel HRP/DAB detection system (PathnSitu Biotechnologies, USA). Images were captured using a brightfield microscope (Olympus, Japan). IHC scoring and percentage positivity were determined using IHC Profiler (4) plug-in in ImageJ software (5).

***In-vitro functional assays:***

***Analysis of cell growth and doubling time:***

Approximately 50,000 cells were seeded in a cell culture dish (35-mm) and cultured for 5 days to analyze their growth pattern. Upon trypsinization at the specified time points, the cells were counted, and their doubling time was calculated using a web tool ([https://doubling-time.com](https://doubling-time.com/)) (6).

***Anchorage-dependent colony formation assay:***

A 6cm plate was seeded with approximately 500 cells and cultured in prescribed media for 14 days. Upon observing distinct colonies, the culture medium was aspirated, and the plate was washed three times with PBS. Then, the colonies were incubated with 0.5% staining solution (crystal violet) for 10 minutes. The stained plate was rinsed with PBS, and colonies were examined and counted under a bright field microscope (Olympus, Japan).

***Cell cycle analysis and Annexin/PI staining:***

The cells were seeded in a culture plate (10 cm) and synchronized with serum starved media for 24 hours, followed by culturing with complete media for another 24 hours. The cells were then trypsinized, fixed overnight at 4°C with 70% ethanol, incubated with RNase-A (Sigma Aldrich, USA) at 37°C for 1 hour, treated with propidium iodide stain (Sigma Aldrich, USA) for approximately 15-30 minutes in the dark at room temperature, measured using flow cytometer (Partec, Germany) and analysed using Cell Quest software (BD Biosciences, USA) (6).

We have performed Annexin/PI staining using HSC-3 cells to investigate the apoptosis upon HOXA9 knockdown. The percentage of apoptotic cells were determined by staining with Annexin/PI, followed by quantification using flowcytometry using FACS Calibur flow cytometer (Partec, Germany) as per manufacturer’s instructions and processed using Cell Quest software (BD Biosciences, USA).

## ***Cell morphological assessment:***

Following HOXA9 knockdown, the morphological alterations in the cells were assessed by F-actin staining. Cells were grown on sterilized coverslips, fixed with paraformaldehyde (4%, Sigma Aldrich, USA) (5 mins), permeabilized with Triton X-100 (0.5% in PBS, Alfa Aesar, USA) (10 mins), blocked with BSA (3% BSA in PBS, Himedia, India) (30 mins), and stained with 0.5μg/mL of Phalloidin-TRITC (Sigma Aldrich, USA) (40 mins) and 1μg/mL of Hoechst (Himedia, India) (10 mins). Mowiol (Sigma Aldrich, USA) was used to mount the coverslip onto a glass slide. The images were captured at 63X magnification using a confocal microscope (SP8-DMi8, Leica Microsystems, Germany) and images were processed using Leica Application Suite software (Leica Microsystems, Germany).

***Migration assay:***

In 6-well plate, the cells were grown until they reach 90% confluency. Upon rinsing with PBS, cells were serum starved for 24–48 hours. Using a 200μL tip, a scratch was made at the centre of the well. Cells were then maintained in complete medium, the rate of migration was visualized at regular time intervals by brightfield microscopy (Olympus, Japan). The rate of cell migration and percentage of wound remaining were calculated using standard formula (6).

***Three-dimensional collagen gel invasion assay:***

Approximately 40,000 cells were seeded per well on a chambered glass slide (Ibidi, Germany). After 200 μL of collagen I mixture (Serva, Germany) was added to each well, the mixture was left to solidify for 2–3 hours at 37°C incubator. Complete media was layered on the collagen gel and incubated for 48 hours. Following aspiration of media and rinsing the gel with PBS, they were fixed (30-45 mins), permeabilized (45 mins), and further blocked (45 mins) at room temperature as described previously. Cells were then stained with Phalloidin-TRITC (90 mins) and Hoechst (15 mins) at room temperature in the dark and then washed with PBS. Images of invading cells were captured at 63X magnification using a confocal microscope (Leica Microsystems, Germany). The 3D images were developed using confocal Z slices, captured at 50–75 μm from the bottom of each well and image processing was done using LAS X (Leica Microsystems, Germany) (6). The average depth of invasion was calculated by analysing the images of the Z-slices.

## ***Immunofluorescence staining:***

The cells were grown on sterile coverslips, fixed, and permeabilized as described earlier. After blocking step, cells were incubated with A-CTNNB1 antibody (1:100, 8814S, CST, USA) overnight at 4°C, followed by secondary antibody (1:1000, FITC-labelled, 31635, Thermo Fisher Scientific, USA) for 2 hours in dark at room temperature. Cells were then counter- stained with Hoechst, rinsed with PBS, and mounted using Mowiol on a clean glass slide. Using laser scanning confocal microscope (Leica Microsystems, Germany), images were captured at 63X magnification (6). The mean florescence intensity was calculated via ImageJ software.

***HOXA9 target prediction and overlap analysis with EMT markers***:

We explored online tools, namely, the Gene Transcription Regulation Database (GTRD) ([http://gtrd.biouml.org](http://gtrd.biouml.org/)) (7) and TFlink (<https://tflink.net/>) (8) which provide HOXA9 targets, based on the TFBS, validated experimentally by ChIP assays. Using the Venny 2.1.0 tool, the common targets were further subjected to overlapping analysis with EMT markers retrieved from the EMTome database ([www.emtome.org](http://www.emtome.org/)) (9,10).

***Mapping the HOXA9 binding sites on its target genes***

We mapped the promoter region of its target genes using GTRD (7) and Animal TFDBv4.0 to determine HOXA9 binding sites (11). Accordingly, four HOXA9 binding sites were found in the vimentin (*VIM*) promoter region, from -1381 bp to 1121 bp upstream of the TSS. The primers were designed to cover the same region (Supplementary Table 10).

***Chromatin immunoprecipitation (ChIP) PCR:***

The HSC-3 cells were treated with formaldehyde for crosslinking, and the chromatin shearing was performed with a sonication system (VCX750, Sonics Inc., USA) at 15 second on/off cycles for 15 minutes to get 100-500 bp fragments. About 1μg of anti-HOXA9 antibody (sc-81291, Santa Cruz, USA) and with the same concentration of nonspecific IgG (Sino Biological, Inc., China) was added to the chromatin mixture and incubated overnight at 4°C. Protein-DNA complexes were captured using protein A/G coupled magnetic beads (Sino Biological, Inc., China) and subsequently washed with different compositions of wash buffers. Then, DNA was isolated via the standard phenol‒chloroform protocol. PCR was performed with 5-10 ng of DNA (12).

***Bisulfite Sanger sequencing and methylation quantification:***

To validate the findings of the MC seq, we performed bisulfite Sanger sequencing using DNA isolated from two normal oral keratinocytes and four OC cell lines. Approximately 250 ng of isolated DNA was processed for bisulfite conversion, using EZ DNA Methylation-Direct™ (Zymo Research, USA). Bisulfite primers for the targeted amplification of the region spanning the *HOXA9* proximal and distal promoters were designed (Supplementary Table 10) using methPrimer (13) and confirmed with the Bisearch tool (14).

PCR was performed using ZymoTaq Premix (Zymo Research, USA) with 1 U HotStart DNA polymerase, and bisulfite-treated DNA (50ng) was used as a template in a Veriti™ thermal Cycler (Thermo Fisher Scientific, USA). Using purified bisulfite PCR products, Sanger sequencing was performed using Genetic Analyzer (ABI 3130, Applied Biosystems™, USA). Visualization and quality control analysis were carried out via BiQAnalyzer (15). Furthermore, cytosine (5mC) for the individual CGs was estimated using the standard formula: Percent mC = [C peak value /(C peak value + T peak value)] × 100 (16).

***Cross-validation of methylation using public datasets and correlation analysis:***

We retrieved OC-TCGA methylation data from the UCSC Xena browser (1), with the methylation levels (β-value), which were quantified using Illumina platform (Human Methylation 450). The methylation of only those CpG probes in the region that presented the highest promoter activity was selected. The average β-values of all the probes were used to determine the mean methylation values of the primary tumor and solid normal tissues. Further, correlation between methylation and expression was determined using the correlation analysis (Pearson or Spearman) based on the normality distribution of datasets.

**References:**

1. Goldman MJ, Craft B, Hastie M, Repecka K, McDade F, Kamath A, et al. Visualizing and interpreting cancer genomics data via the Xena platform. Nat Biotechnol. 2020;38(6):675-8.

2. Ambatipudi S, Gerstung M, Pandey M, Samant T, Patil A, Kane S, et al. Genome-wide expression and copy number analysis identifies driver genes in gingivobuccal cancers. Genes Chromosomes Cancer. 2012;51(2):161-73.

3. Sheu JJ, Lee CC, Hua CH, Li CI, Lai MT, Lee SC, et al. LRIG1 modulates aggressiveness of head and neck cancers by regulating EGFR-MAPK-SPHK1 signaling and extracellular matrix remodeling. Oncogene. 2014;33(11):1375-84.

4. Varghese F, Bukhari AB, Malhotra R, De A. IHC Profiler: an open source plugin for the quantitative evaluation and automated scoring of immunohistochemistry images of human tissue samples. PLoS One. 2014;9(5):e96801.

5. Schneider CA, Rasband WS, Eliceiri KW. NIH Image to ImageJ: 25 years of image analysis. Nat Methods. 2012;9(7):671-5.

6. Bhat S, Adiga D, Shukla V, Guruprasad KP, Kabekkodu SP, Satyamoorthy K. Metastatic suppression by DOC2B is mediated by inhibition of epithelial-mesenchymal transition and induction of senescence. Cell Biol Toxicol. 2022;38(2):237-58.

7. Yevshin I, Sharipov R, Valeev T, Kel A, Kolpakov F. GTRD: a database of transcription factor binding sites identified by ChIP-seq experiments. Nucleic Acids Res. 2017;45(D1):D61-D7.

8. Liska O, Bohar B, Hidas A, Korcsmaros T, Papp B, Fazekas D, et al. TFLink: an integrated gateway to access transcription factor-target gene interactions for multiple species. Database (Oxford). 2022;2022.

9. Cieslik M, Hoang SA, Baranova N, Chodaparambil S, Kumar M, Allison DF, et al. Epigenetic coordination of signaling pathways during the epithelial-mesenchymal transition. Epigenetics Chromatin. 2013;6(1):28.

10. Vasaikar SV, Deshmukh AP, den Hollander P, Addanki S, Kuburich NA, Kudaravalli S, et al. EMTome: a resource for pan-cancer analysis of epithelial-mesenchymal transition genes and signatures. Br J Cancer. 2021;124(1):259-69.

11. Shen WK, Chen SY, Gan ZQ, Zhang YZ, Yue T, Chen MM, et al. AnimalTFDB 4.0: a comprehensive animal transcription factor database updated with variation and expression annotations. Nucleic Acids Res. 2023;51(D1):D39-D45.

12. Bhat S, Kabekkodu SP, Adiga D, Fernandes R, Shukla V, Bhandari P, et al. ZNF471 modulates EMT and functions as methylation regulated tumor suppressor with diagnostic and prognostic significance in cervical cancer. Cell Biol Toxicol. 2021;37(5):731-49.

13. Li LC, Dahiya R. MethPrimer: designing primers for methylation PCRs. Bioinformatics. 2002;18(11):1427-31.

14. Aranyi T, Tusnady GE. BiSearch: ePCR tool for native or bisulfite-treated genomic template. Methods Mol Biol. 2007;402:385-402.

15. Bock C, Reither S, Mikeska T, Paulsen M, Walter J, Lengauer T. BiQ Analyzer: visualization and quality control for DNA methylation data from bisulfite sequencing. Bioinformatics. 2005;21(21):4067-8.

16. Parrish RR, Day JJ, Lubin FD. Direct bisulfite sequencing for examination of DNA methylation with gene and nucleotide resolution from brain tissues. Curr Protoc Neurosci. 2012;Chapter 7:Unit 7 24.
