# Supplementary material for: HOXA9 orchestrates EMT and metastasis in oral cancer via transcriptional activation of vimentin and β-catenin signaling
Source: Cell Death Dis. 2026 Mar 28;17(1):428. doi: 10.1038/s41419-026-08664-7 (PMC13153177; doi:10.1038/s41419-026-08664-7)
Supplement: Supplementary file 2 — Supplementary Tables [file 41419_2026_8664_MOESM2_ESM.docx]

**Supplementary Table 1:** Differential expression of HOXA9 in relation to the demographic and clinicopathological details of the patient samples, analyzed by IHC

|  | **Oral cancer -Clinical samples n (%)** | |  |
| --- | --- | --- | --- |
|  | **HOXA9 expression; IHC O.D score ≥1.25** | **HOXA9 expression; IHC O.D. score<1.25** | **P-value** |
| **Demographic features** | | | |
| **Age at tumor diagnosis** | | | |
| ≤50 (23) | 12 (52.17) | 11 (47.82) | 0.228 |
| >50 (11) | 8 (72.72) | 3 (27.27) |  |
| **Gender** |  |  |  |
| Male (29) | 17 (58.6) | 12 (41.37) | 0.915 |
| Female (6) | 4 (66.66) | 2 (33.33) |  |
| **Clinical features** | | | |
| **Tumor site** | | | |
| Buccal mucosa (14) | 9 (64.28) | 5 (35.71) | 0.483 |
| Gingivo buccal sulcus (5) | 3 (60) | 2 (40) |  |
| Tongue (6) | 2 (33.33) | 4 (66.66) |  |
| Oral cavity (1) | 1 | 0 |  |
| Palate (2) | - | 2 |  |
| Lip and floor of mouth (2) | 2 | 0 |  |
| **Pre-malignant lesions (5)** | 4 (80) | 1 (20) | - |
| **Grade** | | | |
| WDSCC (14) | 9 (64.2) | 5 (35.7) | 0.009 |
| MDSCC (14) | 7 (50) | 7 (50) |  |
| PDSCC (5) | 4 (80) | 1 (20) |  |

**Note:** O.D score: optical density score

**Supplementary Table 2:** KEGG pathways associated with genes downregulated upon HOXA9-knockdown

| **Description** | **Gene count** | **P-value** |
| --- | --- | --- |
| Pathways in cancer | 42 | 0.00001 |
| Focal adhesion | 28 | 0.00007 |
| Regulation of actin cytoskeleton | 28 | 0.00022 |
| Cytokine-cytokine receptor interaction | 27 | 0.01094 |
| Axon guidance | 22 | 0.00002 |
| Cell adhesion molecules | 22 | 0.00005 |
| JAK-STAT signaling pathway | 22 | 0.00033 |
| Osteoclast differentiation | 21 | 0.00006 |
| Amoebiasis | 20 | 0.00001 |
| ECM-receptor interaction | 18 | 0.00001 |
| Tight junction | 18 | 0.00198 |
| Hepatitis C | 18 | 0.00235 |
| Leukocyte transendothelial migration | 16 | 0.00312 |
| Neurotrophin signaling pathway | 16 | 0.00702 |
| Wnt signaling pathway | 16 | 0.03239 |
| Arrhythmogenic right ventricular cardiomyopathy (ARVC) | 15 | 0.00006 |
| ErbB signaling pathway | 15 | 0.00037 |
| Small cell lung cancer | 14 | 0.00093 |
| Hypertrophic cardiomyopathy (HCM) | 13 | 0.00224 |
| Hematopoietic cell lineage | 13 | 0.0038 |
| B cell receptor signaling pathway | 12 | 0.00273 |
| Fc gamma R-mediated phagocytosis | 12 | 0.01796 |
| Melanogenesis | 12 | 0.02776 |
| Chagas disease (American trypanosomiasis) | 12 | 0.03391 |
| T cell receptor signaling pathway | 12 | 0.04354 |
| Basal cell carcinoma | 11 | 0.00061 |
| Complement and coagulation cascades | 11 | 0.00417 |
| Prostate cancer | 11 | 0.02652 |
| Dilated cardiomyopathy | 11 | 0.02854 |
| TGF-beta signaling pathway | 10 | 0.04491 |
| Hedgehog signaling pathway | 9 | 0.00873 |
| Bacterial invasion of epithelial cells | 9 | 0.03696 |
| Leishmaniasis | 9 | 0.0431 |
| Aldosterone-regulated sodium reabsorption | 8 | 0.00463 |
| Malaria | 8 | 0.01511 |
| Arginine and proline metabolism | 8 | 0.02089 |
| NOD-like receptor signaling pathway | 8 | 0.03376 |
| Notch signaling pathway | 7 | 0.02908 |
| Type II diabetes mellitus | 7 | 0.03224 |
| Glycosphingolipid biosynthesis - lacto and neolacto series | 6 | 0.0052 |
| Prion diseases | 6 | 0.0256 |
| Glycosphingolipid biosynthesis - globo series | 4 | 0.01005 |
| Phenylalanine metabolism | 4 | 0.02051 |

**Supplementary Table 3:** KEGG pathways associated with genes upregulated upon HOXA9-knockdown

| **Description** | **Gene Count** | **P-value** |
| --- | --- | --- |
| Neuroactive ligand-receptor interaction | 20 | 0.0004 |
| Nitrogen metabolism | 5 | 0.00068 |
| Long-term depression | 8 | 0.00166 |
| Protein digestion and absorption | 8 | 0.00419 |
| TGF-beta signaling pathway | 8 | 0.00562 |
| Salivary secretion | 8 | 0.0074 |
| Gap junction | 8 | 0.00791 |
| Cell adhesion molecules (CAMs) | 10 | 0.01163 |
| Mucin type O-Glycan biosynthesis | 4 | 0.01462 |
| ECM-receptor interaction | 7 | 0.01873 |
| Amoebiasis | 8 | 0.01998 |
| Long-term potentiation | 6 | 0.02408 |
| Focal adhesion | 12 | 0.02651 |
| Arginine and proline metabolism | 5 | 0.02857 |
| Vascular smooth muscle contraction | 8 | 0.03218 |
| Purine metabolism | 10 | 0.03473 |
| Nicotinate and nicotinamide metabolism | 3 | 0.04004 |
| Riboflavin metabolism | 2 | 0.04651 |
| Glycosaminoglycan biosynthesis - heparan sulfate / heparin | 3 | 0.04911 |

**Supplementary Table 4:** Gene ontology (GO) analysis showing top 30-biological processes (BP) associated with downregulated and upregulated genes upon HOXA9-knockdown

| **Term** | **Category** | **Gene Count** | **P-value** | **Regulation** |
| --- | --- | --- | --- | --- |
| regulation of neuron projection development | BP | 56 | 0.00001 | Down |
| circulatory system process | BP | 56 | 0.00002 | Down |
| blood circulation | BP | 55 | 0.00003 | Down |
| axon development | BP | 55 | 0.00003 | Down |
| regulation of anatomical structure size | BP | 55 | 0.00004 | Down |
| regulation of cell morphogenesis | BP | 53 | 0.00004 | Down |
| regulation of peptidase activity | BP | 52 | 0.00001 | Down |
| T cell activation | BP | 52 | 0.00002 | Down |
| regulation of lymphocyte activation | BP | 52 | 0.00007 | Down |
| negative regulation of transport | BP | 51 | 0.00043 | Down |
| regulation of endopeptidase activity | BP | 50 | 0.00001 | Down |
| axonogenesis | BP | 50 | 0.00008 | Down |
| positive regulation of secretion | BP | 49 | 0.00001 | Down |
| Ras protein signal transduction | BP | 49 | 0.00005 | Down |
| regulation of vasculature development | BP | 48 | 0.00006 | Down |
| muscle system process | BP | 48 | 0.00039 | Down |
| negative regulation of hydrolase activity | BP | 48 | 0.00043 | Down |
| Wnt signaling pathway | BP | 48 | 0.00055 | Down |
| cell-cell signaling by wnt | BP | 48 | 0.00061 | Down |
| positive regulation of secretion by cell | BP | 46 | 0.00001 | Down |
| positive regulation of cytokine production | BP | 46 | 0.00024 | Down |
| positive regulation of establishment of protein localization | BP | 46 | 0.00051 | Down |
| regulation of protein serine/threonine kinase activity | BP | 46 | 0.00628 | Down |
| regulation of actin filament-based process | BP | 45 | 0.00003 | Down |
| positive regulation of neurogenesis | BP | 45 | 0.00191 | Down |
| regulation of vesicle-mediated transport | BP | 45 | 0.01064 | Down |
| ERK1 and ERK2 cascade | BP | 44 | 0.00001 | Down |
| epithelial cell migration | BP | 44 | 0.00001 | Down |
| lipid localization | BP | 44 | 0.00018 | Down |
| positive regulation of protein transport | BP | 44 | 0.00029 | Down |
| signal release | BP | 31 | 0.00001 | Up |
| regulation of neuron projection development | BP | 31 | 0.00003 | Up |
| axon development | BP | 31 | 0.00006 | Up |
| extracellular structure organization | BP | 29 | 0.00001 | Up |
| response to peptide | BP | 29 | 0.00021 | Up |
| calcium ion homeostasis | BP | 28 | 0.00024 | Up |
| cellular divalent inorganic cation homeostasis | BP | 28 | 0.00051 | Up |
| cell growth | BP | 28 | 0.00054 | Up |
| cellular calcium ion homeostasis | BP | 27 | 0.00035 | Up |
| axonogenesis | BP | 27 | 0.00036 | Up |
| regulation of anatomical structure size | BP | 27 | 0.00165 | Up |
| muscle tissue development | BP | 26 | 0.00011 | Up |
| muscle organ development | BP | 26 | 0.00016 | Up |
| response to peptide hormone | BP | 26 | 0.00027 | Up |
| muscle system process | BP | 26 | 0.00101 | Up |
| regulation of cell morphogenesis | BP | 26 | 0.00175 | Up |
| striated muscle tissue development | BP | 25 | 0.00015 | Up |
| modulation of chemical synaptic transmission | BP | 25 | 0.00064 | Up |
| regulation of trans-synaptic signaling | BP | 25 | 0.00066 | Up |
| muscle cell differentiation | BP | 24 | 0.00029 | Up |
| regulation of cell growth | BP | 24 | 0.00133 | Up |
| pattern specification process | BP | 24 | 0.00233 | Up |
| positive regulation of neurogenesis | BP | 24 | 0.004 | Up |
| divalent metal ion transport | BP | 24 | 0.00471 | Up |
| divalent inorganic cation transport | BP | 24 | 0.0051 | Up |
| multicellular organismal homeostasis | BP | 24 | 0.00643 | Up |
| negative regulation of transport | BP | 24 | 0.01144 | Up |
| regulation of cytosolic calcium ion concentration | BP | 23 | 0.00028 | Up |
| eye development | BP | 23 | 0.00029 | Up |
| visual system development | BP | 23 | 0.00031 | Up |

Down- downregulated genes; Up- Upregulated genes.

**Supplementary Table 5:** Gene ontology (GO) analysis showing top 30-molecular functions (MF) associated with downregulated and upregulated genes upon HOXA9-knockdown

| **Description** | **Category** | **Gene Count** | **P-value** | **Regulation** |
| --- | --- | --- | --- | --- |
| channel activity | MF | 55 | 0.00003 | Down |
| passive transmembrane transporter activity | MF | 55 | 0.00003 | Down |
| substrate-specific channel activity | MF | 51 | 0.0001 | Down |
| ion channel activity | MF | 50 | 0.00008 | Down |
| DNA-binding transcription activator activity, RNA polymerase II-specific | MF | 46 | 0.00263 | Down |
| transcription factor activity, RNA polymerase II proximal promoter sequence-specific DNA binding | MF | 45 | 0.00404 | Down |
| metal ion transmembrane transporter activity | MF | 44 | 0.00587 | Down |
| gated channel activity | MF | 42 | 0.00018 | Down |
| phospholipid binding | MF | 41 | 0.00868 | Down |
| cadherin binding | MF | 38 | 0.0005 | Down |
| ion gated channel activity | MF | 38 | 0.00147 | Down |
| enzyme inhibitor activity | MF | 37 | 0.00987 | Down |
| cation channel activity | MF | 35 | 0.00287 | Down |
| cytokine receptor binding | MF | 32 | 0.00227 | Down |
| proximal promoter DNA-binding transcription activator activity, RNA polymerase II-specific | MF | 31 | 0.00979 | Down |
| cytokine activity | MF | 30 | 0.00015 | Down |
| peptidase regulator activity | MF | 28 | 0.00079 | Down |
| serine-type peptidase activity | MF | 28 | 0.01616 | Down |
| serine hydrolase activity | MF | 28 | 0.01924 | Down |
| sulfur compound binding | MF | 27 | 0.00629 | Down |
| protein tyrosine kinase activity | MF | 25 | 0.00031 | Down |
| endopeptidase regulator activity | MF | 25 | 0.00048 | Down |
| actin filament binding | MF | 25 | 0.00091 | Down |
| glycosaminoglycan binding | MF | 25 | 0.00744 | Down |
| serine-type endopeptidase activity | MF | 25 | 0.02901 | Down |
| endopeptidase inhibitor activity | MF | 24 | 0.00063 | Down |
| peptidase inhibitor activity | MF | 24 | 0.00111 | Down |
| secondary active transmembrane transporter activity | MF | 24 | 0.01453 | Down |
| Rab GTPase binding | MF | 23 | 0.00301 | Down |
| integrin binding | MF | 22 | 0.00003 | Down |
| channel activity | MF | 29 | 0.00027 | Up |
| passive transmembrane transporter activity | MF | 29 | 0.00028 | Up |
| ion channel activity | MF | 27 | 0.00032 | Up |
| substrate-specific channel activity | MF | 27 | 0.00055 | Up |
| receptor ligand activity | MF | 26 | 0.00352 | Up |
| metal ion transmembrane transporter activity | MF | 25 | 0.00267 | Up |
| ion gated channel activity | MF | 22 | 0.0009 | Up |
| gated channel activity | MF | 22 | 0.00126 | Up |
| actin binding | MF | 22 | 0.01079 | Up |
| cell adhesion molecule binding | MF | 22 | 0.0447 | Up |
| cation channel activity | MF | 20 | 0.00213 | Up |
| nucleoside-triphosphatase regulator activity | MF | 18 | 0.02465 | Up |
| enzyme inhibitor activity | MF | 18 | 0.03753 | Up |
| carbohydrate binding | MF | 17 | 0.00398 | Up |
| GTPase regulator activity | MF | 17 | 0.01482 | Up |
| metallopeptidase activity | MF | 16 | 0.00024 | Up |
| anion transmembrane transporter activity | MF | 16 | 0.04814 | Up |
| G protein-coupled receptor binding | MF | 15 | 0.02208 | Up |
| GTPase activator activity | MF | 15 | 0.02543 | Up |
| sulfur compound binding | MF | 14 | 0.01736 | Up |
| DNA-binding transcription repressor activity, RNA polymerase II-specific | MF | 14 | 0.03611 | Up |
| metalloendopeptidase activity | MF | 13 | 0.00004 | Up |
| extracellular matrix structural constituent | MF | 13 | 0.00098 | Up |
| cytokine activity | MF | 13 | 0.0177 | Up |
| glycosaminoglycan binding | MF | 13 | 0.01957 | Up |
| proximal promoter DNA-binding transcription repressor activity, RNA polymerase II-specific | MF | 12 | 0.00593 | Up |
| secondary active transmembrane transporter activity | MF | 12 | 0.04235 | Up |
| growth factor binding | MF | 11 | 0.0033 | Up |
| oxidoreductase activity, acting on paired donors, with incorporation or reduction of molecular oxygen | MF | 11 | 0.01083 | Up |
| protein C-terminus binding | MF | 11 | 0.02937 | Up |

**Supplementary Table 6:** Gene ontology (GO) analysis showing top 30-cellular components (CC) associated with downregulated and upregulated genes upon HOXA9-knockdown

| **Description** | **Category** | **Count** | **P-value** | **Regulation** |
| --- | --- | --- | --- | --- |
| cytoplasmic region | CC | 44 | 0.00809 | Down |
| cell leading edge | CC | 39 | 0.00305 | Down |
| membrane region | CC | 37 | 0.00022 | Down |
| membrane raft | CC | 36 | 0.00021 | Down |
| membrane microdomain | CC | 36 | 0.00022 | Down |
| cell cortex | CC | 35 | 0.0002 | Down |
| endoplasmic reticulum lumen | CC | 35 | 0.00036 | Down |
| cell-substrate junction | CC | 35 | 0.04175 | Down |
| extrinsic component of membrane | CC | 33 | 0.00064 | Down |
| basolateral plasma membrane | CC | 31 | 0.00001 | Down |
| transmembrane transporter complex | CC | 31 | 0.0145 | Down |
| transporter complex | CC | 31 | 0.01977 | Down |
| ion channel complex | CC | 29 | 0.01556 | Down |
| endocytic vesicle | CC | 28 | 0.01898 | Down |
| intermediate filament cytoskeleton | CC | 26 | 0.00508 | Down |
| intermediate filament | CC | 25 | 0.00105 | Down |
| extrinsic component of plasma membrane | CC | 24 | 0.00008 | Down |
| actin-based cell projection | CC | 24 | 0.00163 | Down |
| plasma membrane receptor complex | CC | 23 | 0.00134 | Down |
| lamellipodium | CC | 21 | 0.0079 | Down |
| contractile fiber part | CC | 21 | 0.02986 | Down |
| ruffle | CC | 19 | 0.00924 | Down |
| cell cortex part | CC | 19 | 0.00982 | Down |
| cytoplasmic side of plasma membrane | CC | 18 | 0.00607 | Down |
| specific granule | CC | 18 | 0.01236 | Down |
| tertiary granule | CC | 18 | 0.01563 | Down |
| recycling endosome | CC | 18 | 0.0185 | Down |
| anchored component of membrane | CC | 18 | 0.02063 | Down |
| cytoplasmic side of membrane | CC | 18 | 0.02417 | Down |
| vacuolar lumen | CC | 17 | 0.04248 | Down |
| neuronal cell body | CC | 29 | 0.00019 | Up |
| receptor complex | CC | 27 | 0.00004 | Up |
| neuron to neuron synapse | CC | 25 | 0.00002 | Up |
| postsynaptic membrane | CC | 24 | 0.00003 | Up |
| postsynaptic specialization | CC | 23 | 0.00016 | Up |
| cytoplasmic region | CC | 23 | 0.01303 | Up |
| ion channel complex | CC | 22 | 0.00007 | Up |
| postsynaptic density | CC | 22 | 0.00015 | Up |
| asymmetric synapse | CC | 22 | 0.00018 | Up |
| transmembrane transporter complex | CC | 22 | 0.00022 | Up |
| collagen-containing extracellular matrix | CC | 22 | 0.0003 | Up |
| transporter complex | CC | 22 | 0.00031 | Up |
| endoplasmic reticulum lumen | CC | 21 | 0.0002 | Up |
| axon part | CC | 20 | 0.00647 | Up |
| cell-cell junction | CC | 20 | 0.03395 | Up |
| glutamatergic synapse | CC | 19 | 0.00691 | Up |
| apical part of cell | CC | 19 | 0.01301 | Up |
| apical plasma membrane | CC | 18 | 0.00401 | Up |
| distal axon | CC | 16 | 0.00793 | Up |
| membrane raft | CC | 16 | 0.01635 | Up |
| membrane microdomain | CC | 16 | 0.01681 | Up |
| membrane region | CC | 16 | 0.02257 | Up |
| cell projection membrane | CC | 16 | 0.03506 | Up |
| sarcomere | CC | 15 | 0.00065 | Up |
| contractile fiber part | CC | 15 | 0.00151 | Up |
| myofibril | CC | 15 | 0.00181 | Up |
| cation channel complex | CC | 15 | 0.00189 | Up |
| contractile fiber | CC | 15 | 0.0029 | Up |
| cell cortex | CC | 15 | 0.02383 | Up |
| sarcolemma | CC | 12 | 0.0005 | Up |

**Supplementary Table 7:** Prediction of targets of HOXA9 using experimentally validated databases and its overlapping analysis with EMT markers

| **Transcription factor** | **Targets involved in EMT** |
| --- | --- |
| HOXA9 | ESR1, BIRC3, COL5A1, CDH2, FHL1, KRT7, INHBA, CD36, DLG1, MITF, DESI1, NLK, CLDN4, MTA3, COL5A2, VIM, OCLN, PTP4A1, EGF, TCF4, CD47, VPS13A, PTK2, ZEB2 |

| **Clinical samples n (%)** | | | |
| --- | --- | --- | --- |
|  | **Pre-cancerous (n=25)** | **Node-negative (n=25)** | **Node-positive (n=25)** |
| **Demographic features** | | | |
| **Age at tumor diagnosis** (Mean±SD) | 61±12.6 | 58.52±11.42 | 54.56±14.74 |
| ≤50 | 5 (20) | 8 (32) | 10 (40) |
| >50 | 20 (80) | 17 (68) | 15 (60) |
| **Gender** |  |  |  |
| Male | 19 (76) | 18 (72) | 18 (72) |
| Female | 6 (24) | 7 (28) | 7 (28) |
| **Risk factors** |  |  |  |
| Alcohol | 1 (4) | 1 (4) | 0 |
| Tobacco | 7 (28) | 9 (36) | 10 (40) |
| Tobacco and alcohol | 3 (12) | 2 (8) | 1 (4) |
| Tobacco and smoking | 1 (4) | 0 | 1 (4) |
| Betelnut/Gutka/arecanut chewing | 3 (12) | 4 (16) | 6 (24) |
| None | 10 (40) | 9 (36) | 7 (28) |
| **Tumor site** |  |  |  |
| Buccal mucosa | 14 (63.6) | 15 (65.21) | 11 (44) |
| Gingivo buccal sulcus | 1 (4.54) | 1 (4.34) | 3 (12) |
| Tongue | 6 (27.2) | 5 (21.7) | 8 (32) |
| Alveolus | 1 (4.54) | 1 (4.34) | 2 (8) |
| Maxilla | 0 | 0 | 1 (4) |
| Floor of mouth | 0 | 1 (4.34) | 0 |
| **Pathological parameters** | | | |
| **Clinical stage** |  |  |  |
| Stage I | - | 5 (20) | - |
| Stage II | - | 14 (56) | - |
| Stage III | - | 6 (24) | 6 (24) |
| Stage IV | - | - | 19 (76) |
| **Histological grades** | | | |
| WDSCC | - | 14 (56) | 17 (68) |
| MDSCC | - | 11 (44) | 7 (28) |
| PDSCC | **-** | **-** | 1 (4) |

**Supplementary Table 8:** Demographic and clinical characteristics of the study participants in the present study

|  | **Source** | **Normal** | **Pre-cancerous** | **Tumor** | **Cell Lines** | **Total Samples** |
| --- | --- | --- | --- | --- | --- | --- |
| **Our data (RNA seq)** | Collected | 22 | 7 | 15 | **-** | 44 |
| **Our data (qRT-PCR)** | Collected | 75 (matched normal) | 25 | 50 | 6 | 156 |
| **GSE23558** | GEO dataset | 5 | - | 27 | - | 32 |
| **GSE37991** | GEO dataset | 40 | - | 40 | - | 80 |
| **GDC-TCGA (HNSC)** | UCSC Xena | 44 | - | 520 | - | 564 |
| **Total Samples** |  | 186 | 32 | 652 | 6 | **876** |

**Supplementary Table 9:** Number of data sets and samples analyzed in the present study

**Supplementary Table 10:** List of primers used in the study

| **Primer Name** | **Sequence (5'-3')** | **Annealing Temperature** | **Product Size** |
| --- | --- | --- | --- |
| **Bisulfite Genomic Sequencing (BGS)** |  |  |  |
| HOXA9-core-1-F | TTTGTGTGGTTTTTGAAATAATAATTTT | 58^o^C | 263bp |
| HOXA9-core-1-R | AATCAAATCTAACCTTATCTCTATACTCTC |  |  |
| HOXA9-core-2-F | AGAGTATAGAGATAAGGTTAGATTTGATTT | 58^o^C | 373bp |
| HOXA9-core-2-R | AATAATTACCCAAAACCCCAATAATA |  |  |
| HOXA9-distal-1-F | TTTTTAGGAATATTTTATTGGGGTTTGT | 54^o^C | 264bp |
| HOXA9-distal-1-R | AAAAAAAATAAATCCCAACAACAAAAAA |  |  |
| HOXA9-distal-2-F | GATATTGTTTTTATTATTTTAGTTGAAAT | 58^o^C | 354bp |
| HOXA9-distal-2-R | ATAACAAAAAATCCCTAAACAAAC |  |  |
| **Promoter Construct: Luciferase Assay** |  |  |  |
| HOXA9-Luc-F | ATTAAGATCTCCAGCTGATGAGAAAGGC | 58^o^C | 983bp |
| HOXA9-Luc-R | GGGGAAGCTTAAGTACAGTCACCTAATA |  |  |
| HOXA9-DC1-Luc-F | TCCGGTACCTGCGTTGAATTTGACT | 52^o^C | 684bp |
| HOXA9-DC1-Luc-R | GGGGAAGCTTAAGTACAGTCACCTAATA |  |  |
| HOXA9-DC2-Luc-F | GCTGGGTACCCTTTTTTATGGCTTC | 52^o^C | 409bp |
| HOXA9-DC2-Luc-R | GGGGAAGCTTAAGTACAGTCACCTAATA |  |  |
| HOXA9-DC3-Luc-F | TTAAGGTACCACAAACCCCATCGTAG | 52^o^C | 204bp |
| HOXA9-DC3-Luc-R | GGGGAAGCTTAAGTACAGTCACCTAATA |  |  |
| **ChIP-PCR** |  |  |  |
| VIM - F | CCTTTAATGACTTCCACCAG | 56°C | 128bp |
| VIM - R | CATGAAACCACACCCAAC |  |  |
| Note: The sequence underlined within the table represents the restriction sites incorporated for cloning;  Distal promoter constructs were designed and procured from Synbio Technologies, USA | | | |

| **Promoter** | **Promoter constructs** | **Spanning Region (relative to TSS)** |
| --- | --- | --- |
| HOXA9 proximal promoter | Full length promoter | -989 bp to -6 bp |
|  | Deletion construct-1 | -690 bp to -6 bp |
|  | Deletion construct-2 | -415 bp to -6 bp |
|  | Deletion construct-3 | -210 bp to -6 bp |
| HOXA9 distal promoter | Full length promoter | -4961 bp to -3956 bp |
|  | Deletion construct-1 | -4311 bp to -3761 bp |
|  | Deletion construct-2 | -4011 bp to -3761 bp |
|  | Deletion construct-3 | -2315 bp and -1703bp |

**Supplementary Table 11:** List of promoter constructs designed for dual luciferase reporter assay
