# Supplementary material for: HOXA9 orchestrates EMT and metastasis in oral cancer via transcriptional activation of vimentin and β-catenin signaling
Source: Cell Death Dis. 2026 Mar 28;17(1):428. doi: 10.1038/s41419-026-08664-7 (PMC13153177; doi:10.1038/s41419-026-08664-7)
Supplement: Supplementary file 3 — Supplementary Figures [file 41419_2026_8664_MOESM3_ESM.docx]

**Supplementary Figure 1:**

**
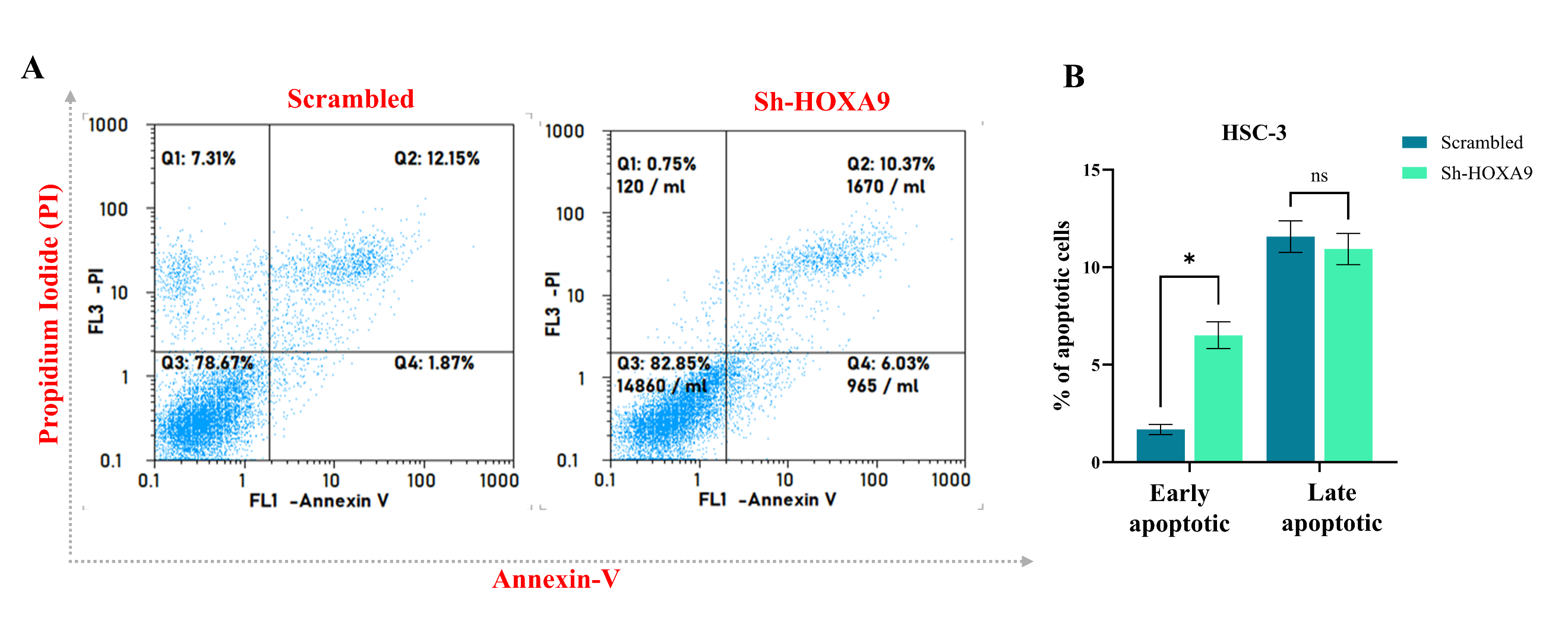
Supplementary Fig.1. Analysis of apoptosis upon HOXA9 knockdown via Annexin/PI staining. (A)** Representative scatter plot images showing percentage of apoptotic cells in scrambled and HOXA9-knockdown HSC-3 cells. **(B)** Quantitative analysis showing percentage of Annexin-V positive cells significantly higher in HOXA9 knockdown cells compared to scrambled. (Early apoptotic: Scr v/s Sh-HOXA9: 1.68%±0.26 v/s 6.51%±0.68)

**Supplementary Figure 2:**

**
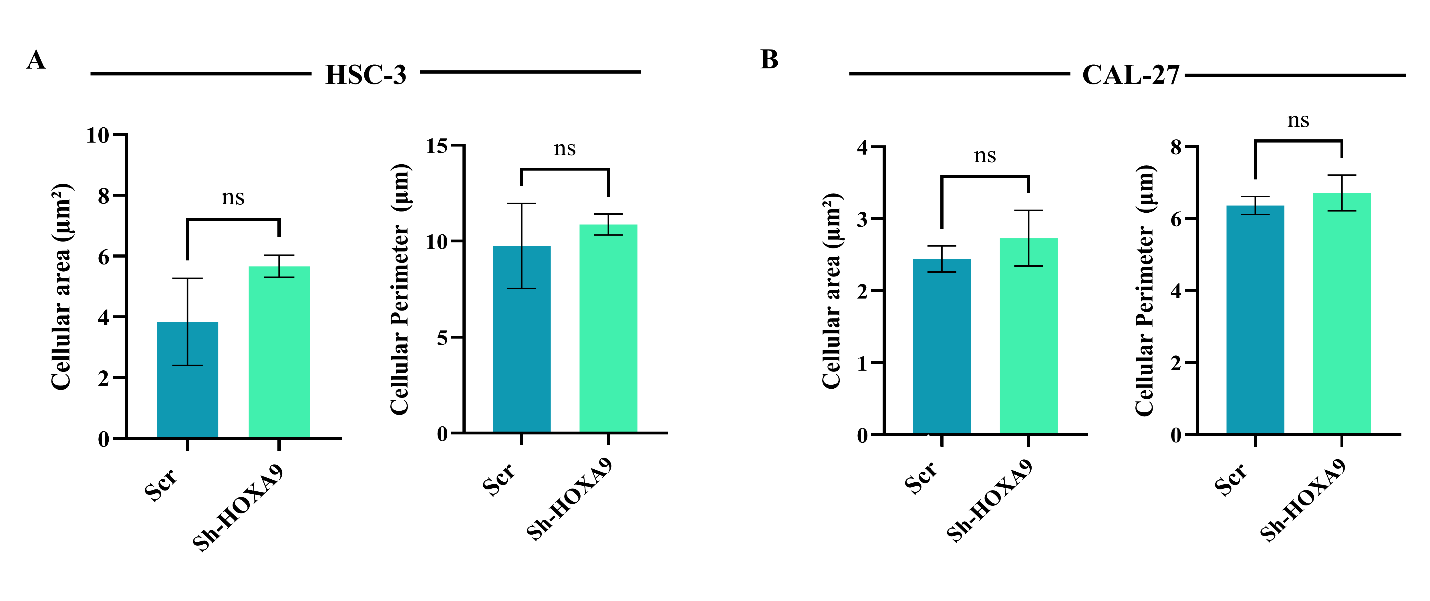
**

**Supplementary Fig.2. Quantification of actin-phalloidin stained images.** Quantitative estimation of **(A)** cellular area and **(B)** cellular perimeter of actin-phalloidin stained images of scrambled and HOXA9-knockdown cells, represented as bar plots. The analysis showed increased cellular area in knockdown cells (HSC-3: 5.66μm^2^±0.36; CAL-27: 2.73μm^2^±0.38) compared to scrambled (HSC-3: 3.83μm^2^±1.43; CAL-27: 2.44μm^2^±0.18), accompanied by larger cellular perimeter.

**Supplementary Figure 3:**

**
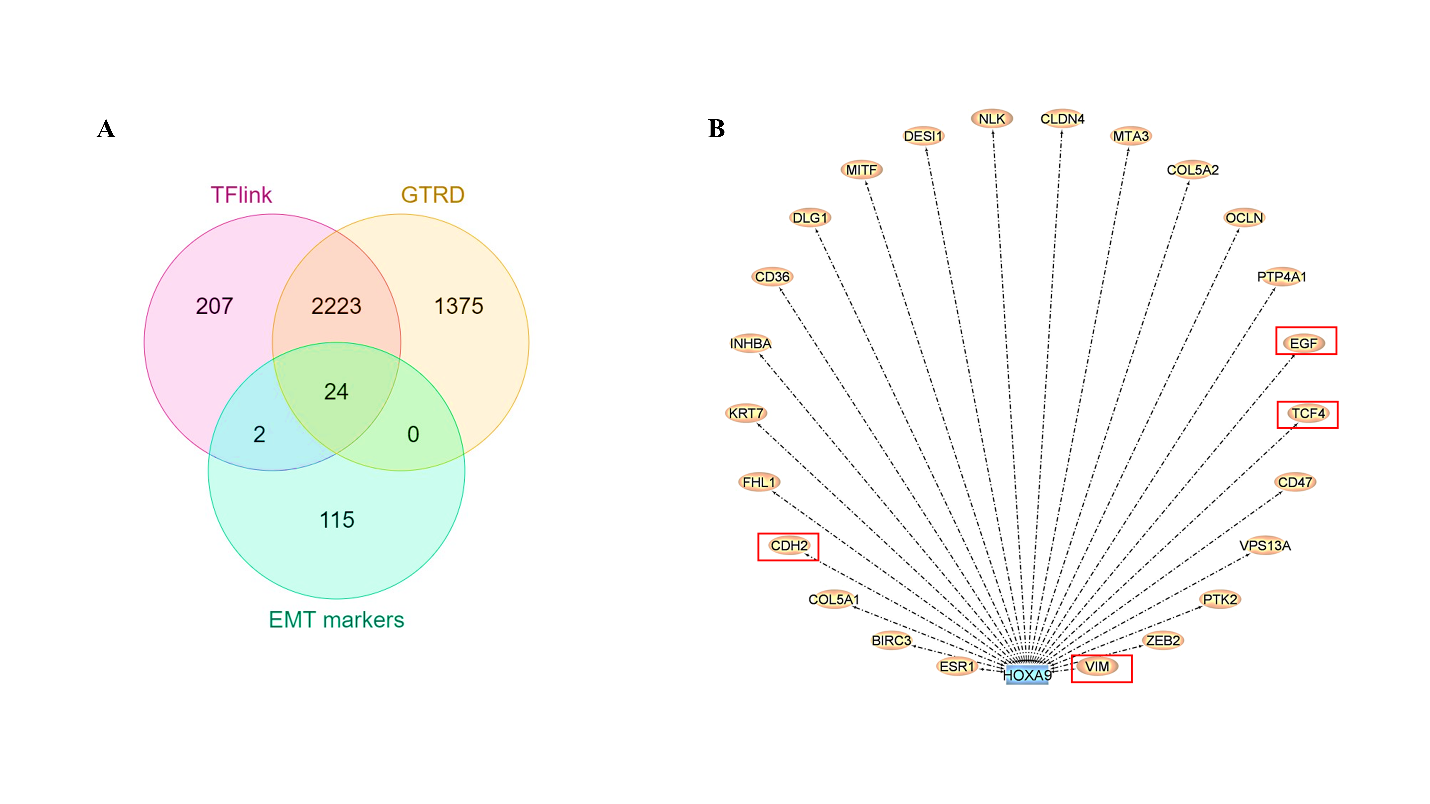
**

**Supplementary Fig.3. Identification of targets of HOXA9 using computational tools.** **(A)** Overlapping analysis of targets of HOXA9 with EMT markers using Venny 2.1.0 tool. **(B)** Cytoscape network shows the targets of HOXA9 transcription factor involved in EMT.

**Supplementary Figure 4:**


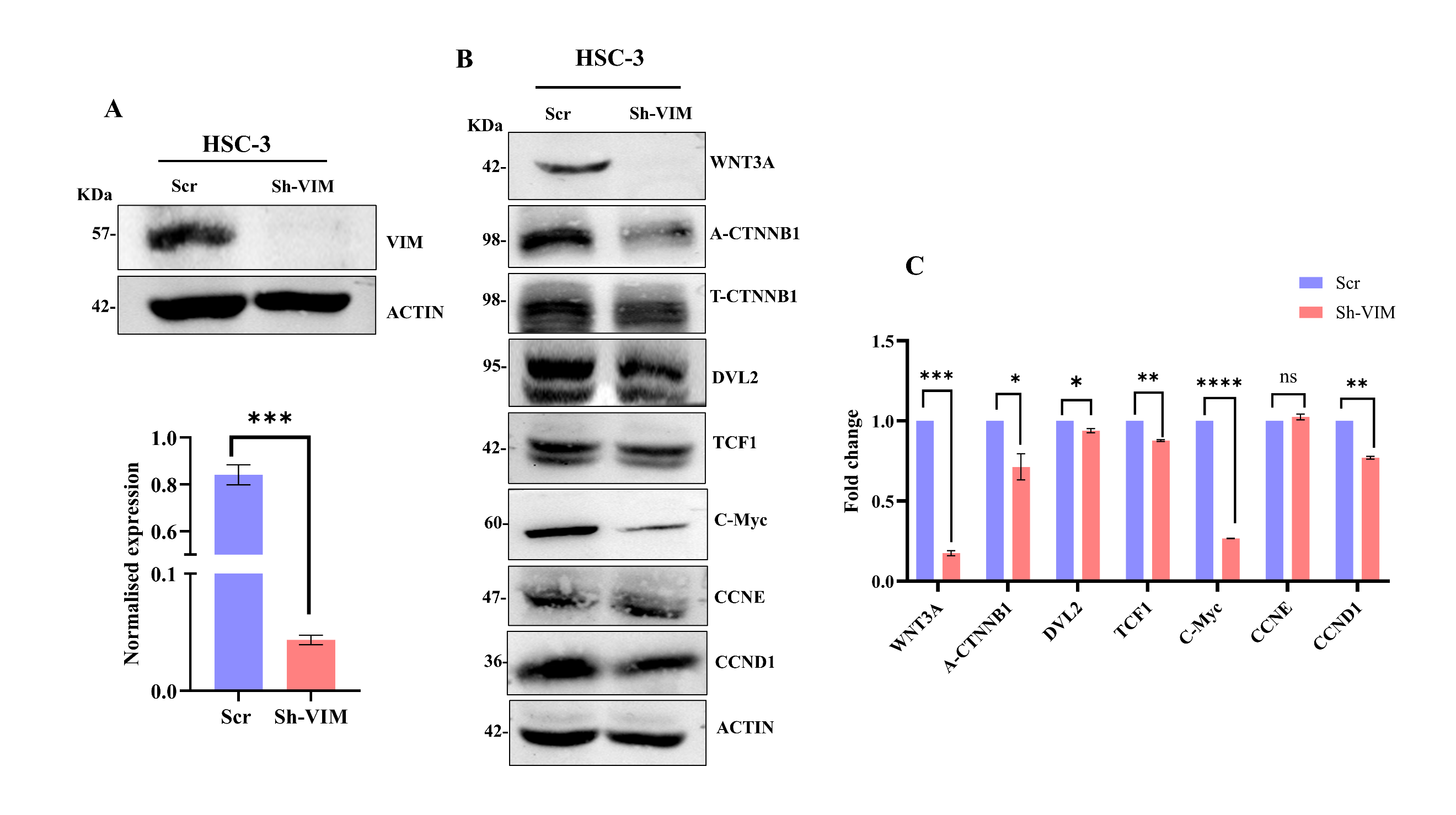
**Supplementary Fig.4. Effect of knockdown of VIM in HSC-3 cells. (A)** Representative western blot images and its quantitative estimation (bar graphs) showing efficient knockdown of VIM in HSC-3 cells. **(B) &(C)** Western blot images with their quantitative analysis (bar graphs) of proteins of Wnt/β-catenin pathway. Knockdown of VIM leads to concomitant reduction in the protein levels of WNT3A, A-CTNNB1, DVL2 and TCF1 and its targets (CCND1 and C-Myc) in HSC-3 cells.

**Supplementary Figure 5:**


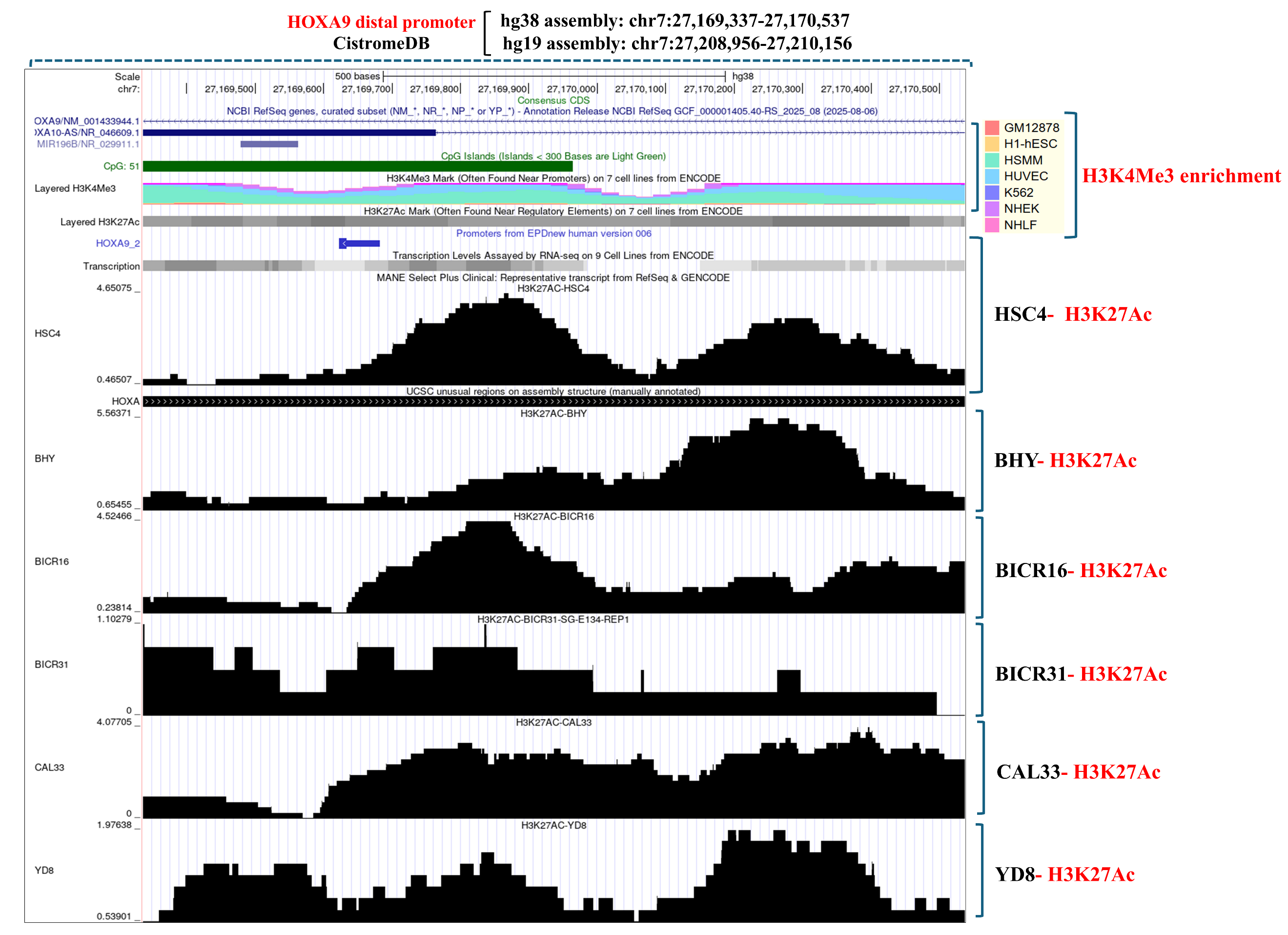


**Supplementary Fig.5. Data retrieval and visualization of *HOXA9* distal promoter.** Representative image shows H3K4Me3 enrichment in seven different cell lines and H3K27Ac deposition on *HOXA9* distal promoter in 6 different OC cell lines (data curated from ENCODE), visualized in UCSC genome browser, from hg38 assembly. Enrichment of activating histone marks H3K27Ac was observed on the distal promoter in HSC-4 (Tongue SCC), YD-8 (Tongue SCC), BHY (Alveolus SCC), CAL-33 (Tongue SCC), BICR31 and BICR16 (Tongue SCC) cell lines. However, H3K4Me3 has also been associated with distal promoter region in GM12878 (lymphoblastoid), H1-hESC (human embryonic stem cells), HSMM (human skeletal muscle myoblasts), HUVEC (human umbilical vein endothelial cells), K562 (erythroleukemic), NHEK (normal human epidermal keratinocytes), and NHLF (normal human lung fibroblasts)**.** However, the data for H3K4Me3 modifications were not available for OC cell lines.
