# Supplementary material for: HOXA9 orchestrates EMT and metastasis in oral cancer via transcriptional activation of vimentin and β-catenin signaling
Source: Cell Death Dis. 2026 Mar 28;17(1):428. doi: 10.1038/s41419-026-08664-7 (PMC13153177; doi:10.1038/s41419-026-08664-7)
Supplement: Supplementary file 4 — Uncropped Western blot [file 41419_2026_8664_MOESM4_ESM.pdf]

Fig.2B:

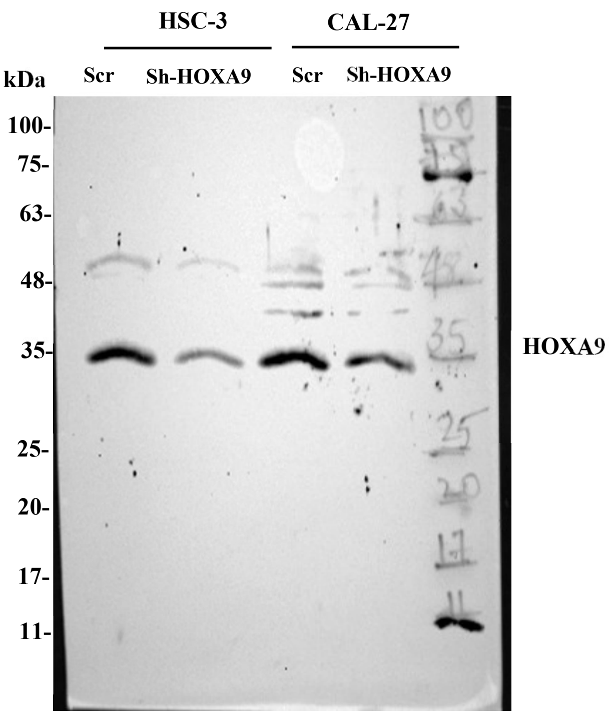

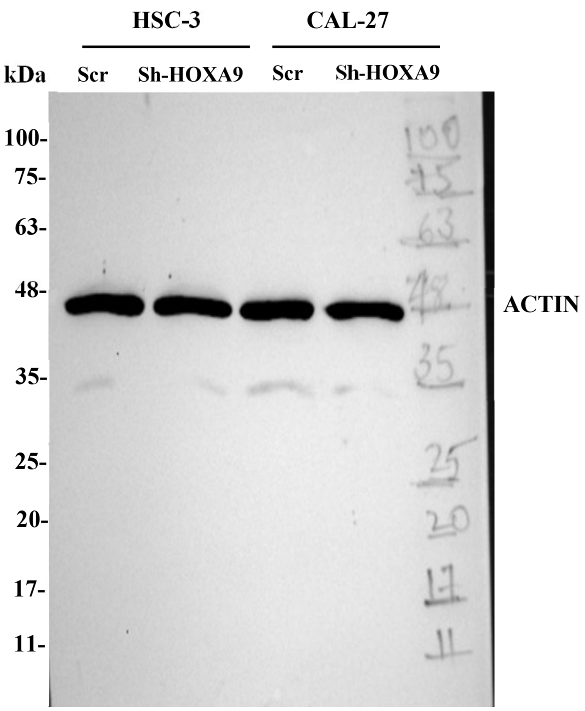

Captured using:

iBright 1500 (Invitrogen, USA)

Fig.6A:

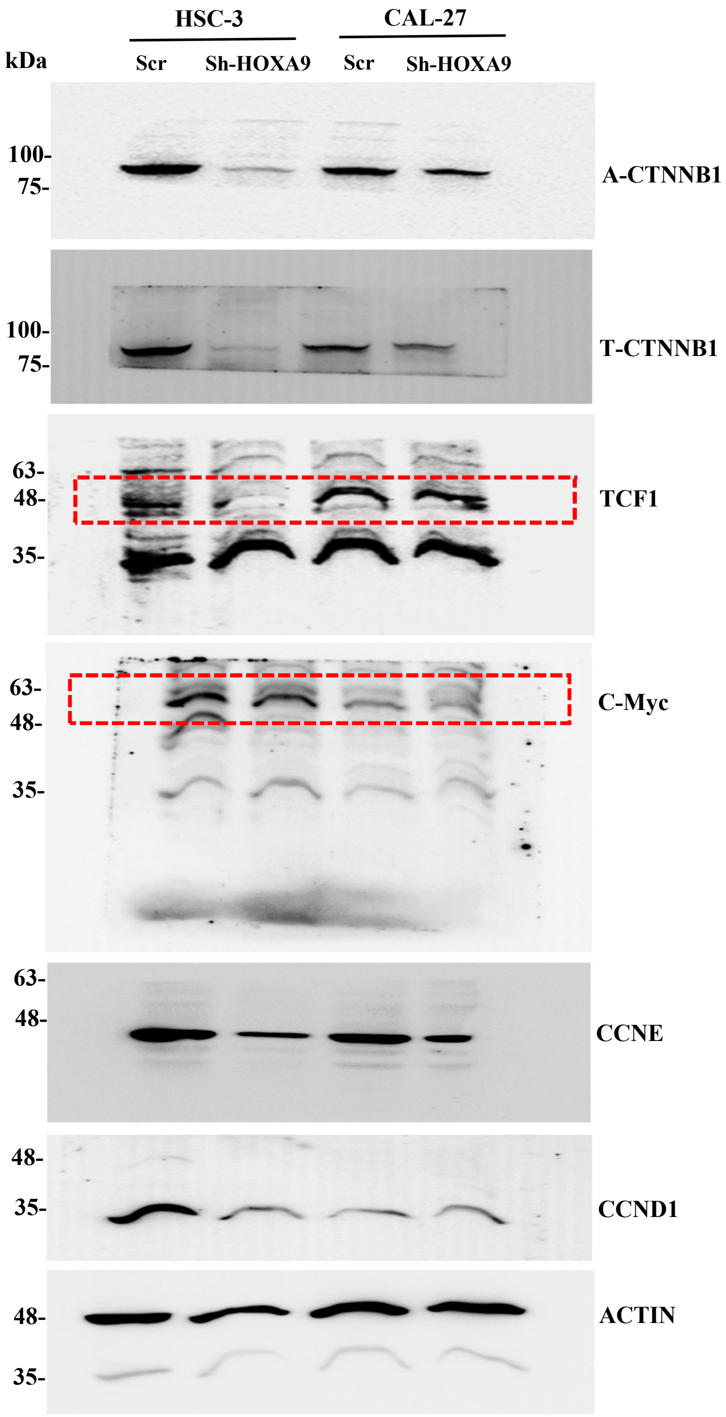

Captured using:

Image Quant LAS 4000 (GE Healthcare, USA)

Fig.6B:

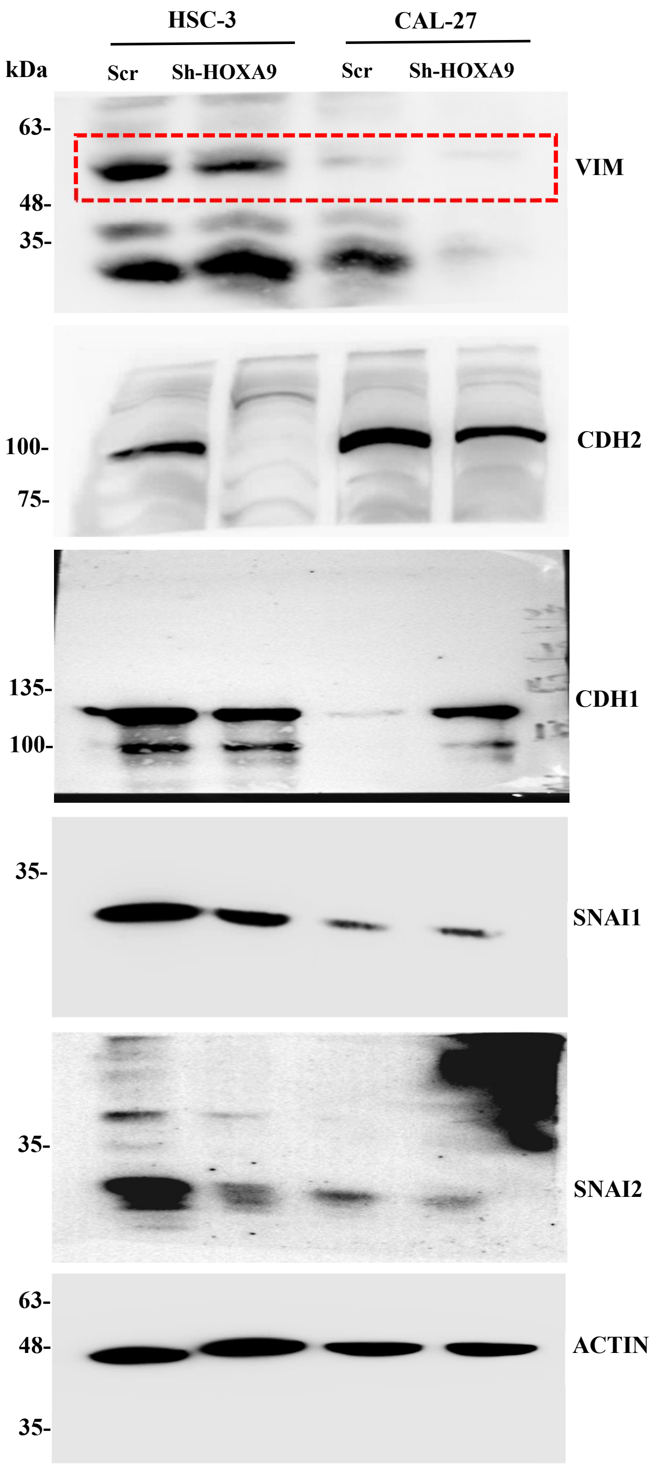

Captured using:

iBright 1500 (Invitrogen, USA)

Image Quant LAS 4000 (GE Healthcare, USA)

Fig.6C:

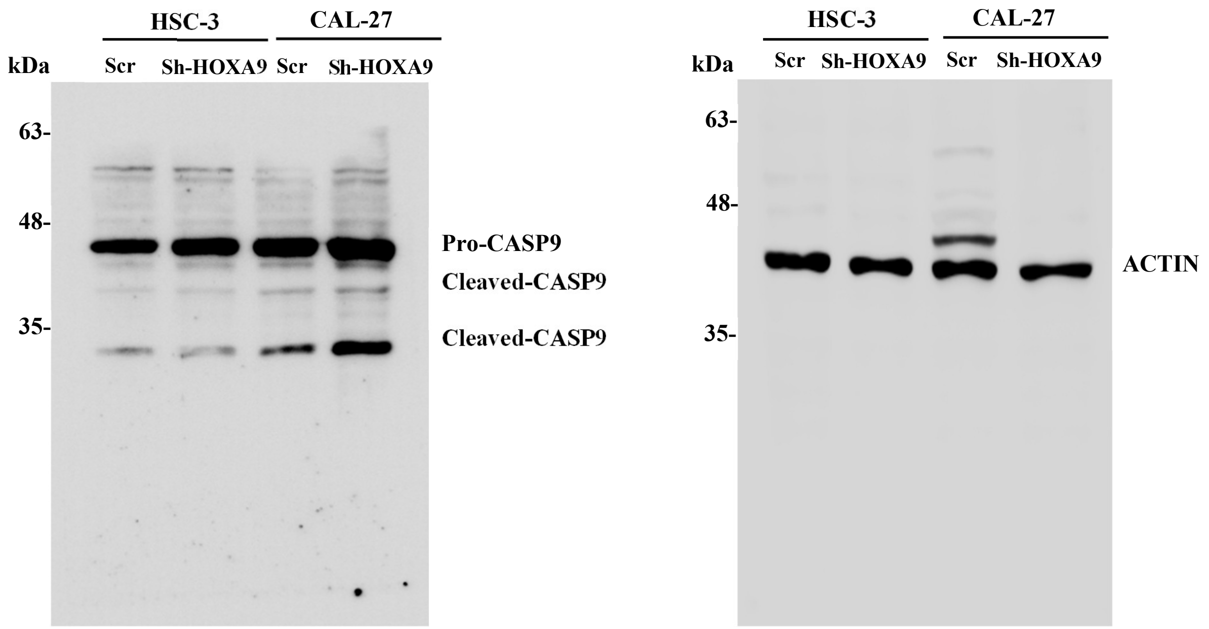

Captured using:

Image Quant LAS 4000 (GE Healthcare, USA)

Fig.6D:

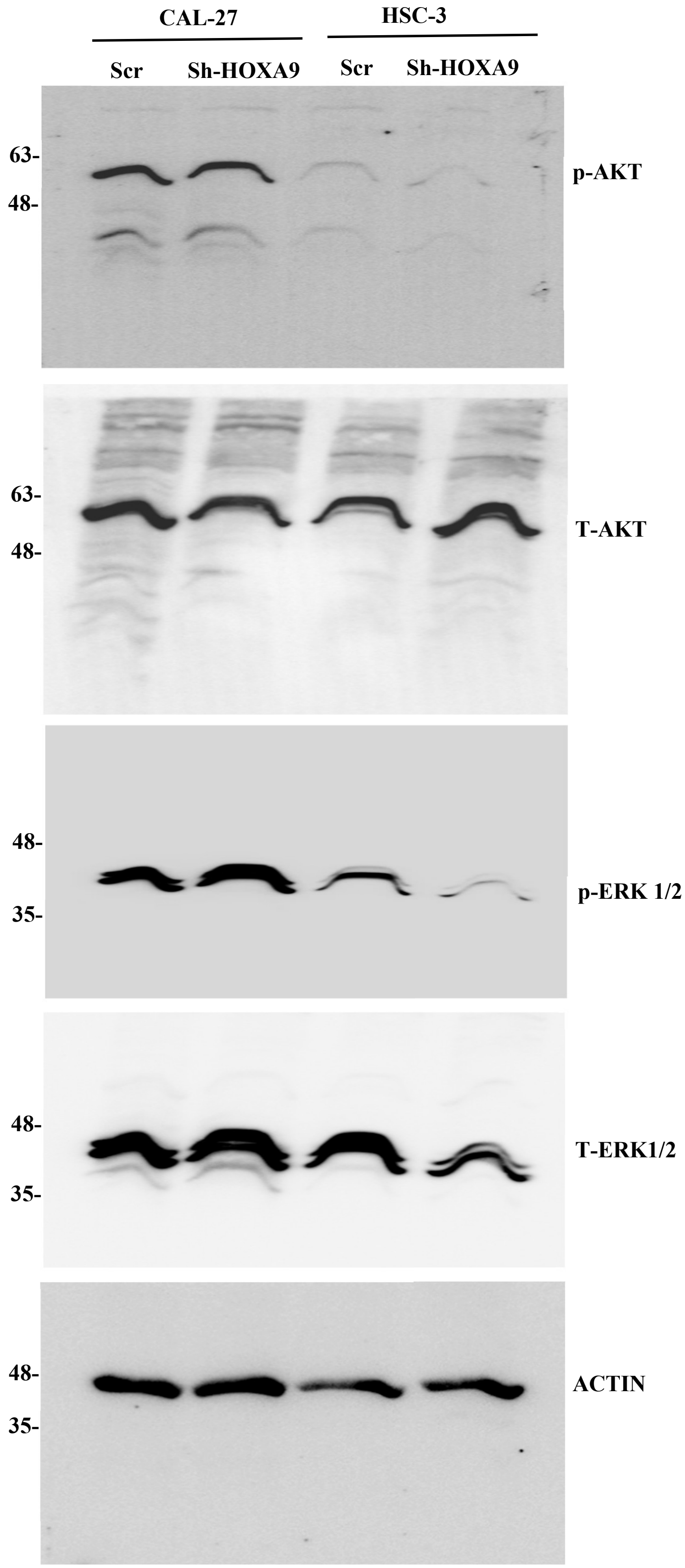

Captured using:

Image Quant LAS 4000 (GE Healthcare, USA)

Fig.6H:

Uncropped gel image of ChIP PCR:

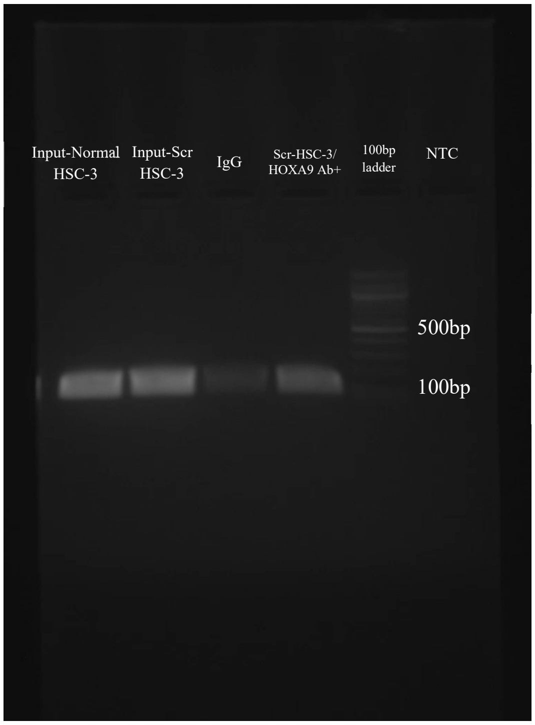

Lane 1: Input-Normal HSC-3 cells
Lane 2: Input- Scrambled HSC-3 cells
Lane 3: Negative control- Scrambled HSC-3 cells immunoprecipitated with IgG antibody
Lane 4: Scrambled HSC-3 cells immunoprecipitated with HOXA9 antibody
Lane 5: 100bp ladder
Lane 6: Negative control

Supplementary Fig.4A:

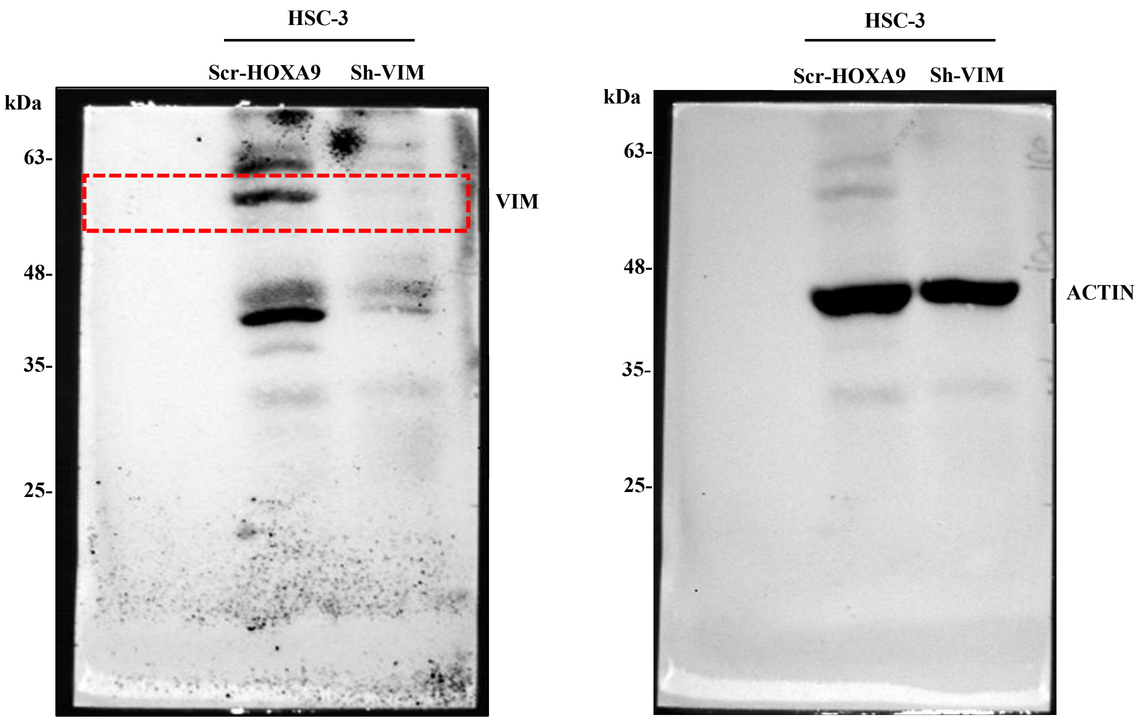

Captured using:

iBright 1500 (Invitrogen, USA)

Supplementary Fig.4B:

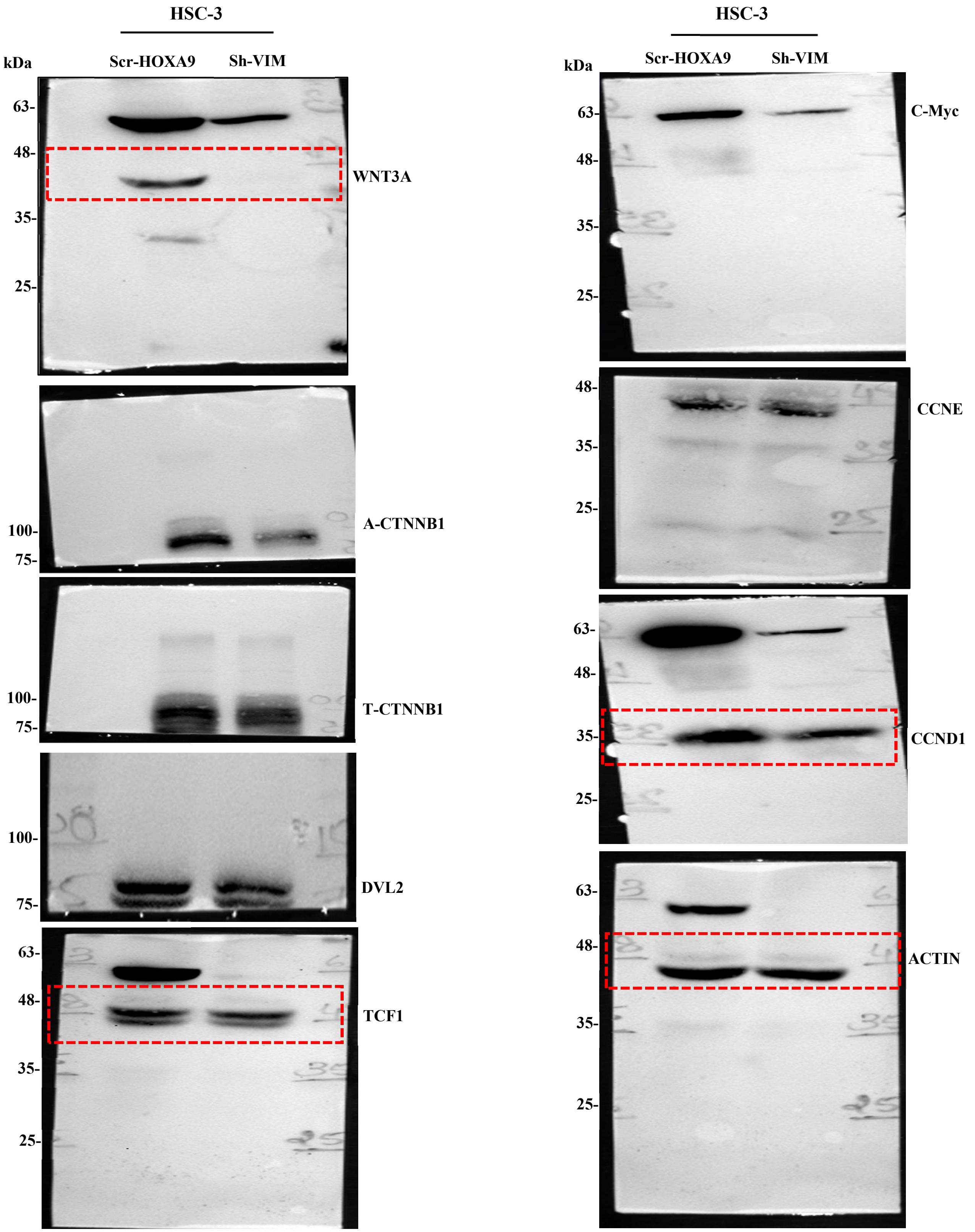

Captured using:

iBright 1500 (Invitrogen, USA)
